# Defense mechanisms against herbivory in *Picea*: sequence evolution and expression regulation of gene family members in the phenylpropanoid pathway

**DOI:** 10.1186/1471-2164-12-608

**Published:** 2011-12-16

**Authors:** Ilga Porth, Björn Hamberger, Richard White, Kermit Ritland

**Affiliations:** 1 Department of Forest Sciences, University of British Columbia, 2424 Main Mall, Vancouver, BC V6T1Z4, Canada; 2 Michael Smith Laboratories, University of British Columbia, 2185 East Mall, Vancouver, BC V6T1Z4, Canada; 3 Department of Statistics, University of British Columbia, 6356 Agricultural Road, Vancouver, BC V6T1Z2, Canada

## Abstract

**Background:**

In trees, a substantial amount of carbon is directed towards production of phenolics for development and defense. This metabolic pathway is also a major factor in resistance to insect pathogens in spruce. In such gene families, environmental stimuli may have an important effect on the evolutionary fate of duplicated genes, and different expression patterns may indicate functional diversification.

**Results:**

Gene families in spruce (*Picea*) have expanded to superfamilies, including O-methyltransferases, cytochrome-P450, and dirigents/classIII-peroxidases. Neo-functionalization of superfamily members from different clades is reflected in expression diversification. Genetical genomics can provide new insights into the genetic basis and evolution of insect resistance in plants. Adopting this approach, we merged genotype data (252 SNPs in a segregating pedigree), gene expression levels (for 428 phenylpropanoid-related genes) and measures of susceptibility to *Pissodes stobi*, using a partial-diallel crossing-design with white spruce (*Picea glauca*). Thirty-eight expressed phenylpropanoid-related genes co-segregated with weevil susceptibility, indicating either causative or reactive effects of these genes to weevil resistance. We identified eight regulatory genomic regions with extensive overlap of quantitative trait loci from susceptibility and growth phenotypes (pQTLs) and expression QTL (eQTL) hotspots. In particular, SNPs within two different CCoAOMT loci regulate phenotypic variation from a common set of 24 genes and three resistance traits.

**Conclusions:**

Pest resistance was associated with individual candidate genes as well as with trans-regulatory hotspots along the spruce genome. Our results showed that specific genes within the phenylpropanoid pathway have been duplicated and diversified in the conifer in a process fundamentally different from short-lived angiosperm species. These findings add to the information about the role of the phenylpropanoid pathway in the evolution of plant defense mechanisms against insect pests and provide substantial potential for the functional characterization of several not yet resolved alternative pathways in plant defenses.

## Background

Lignin, the structural component of land plants, is a heteropolymer of coupled phenylpropanoid monomers derived from hydroxycinnamyl alcohols; it is also the second most abundant biopolymer after cellulose on earth. In gymnosperms, 30% of wood dry weight is lignin, in angiosperms this value is up to 25% ([1,2]). Lignocellulosic polymers are the dominant carbon sink in forest ecosystems and account for approximately 20% of the terrestrial carbon storage [3]. In addition, the phenylpropanoid pathway synthesizes a plethora of specialized plant products (anthocyanins, flavonoids, condensed tannins, stilbenes, soluble and cell wall-bound phenolics, and other polyphenols) with protective functions (antioxidant [4], radical scavenging [5], stress induction [6], UV [7], flavonoids and UV tolerance [8,9]). The biosynthesis of those phenylpropanoids involves intricate networks [10] as shown by the KEGG pathways http://www.genome.jp/kegg/pathway/map/map00940.html.

**Figure 1:**
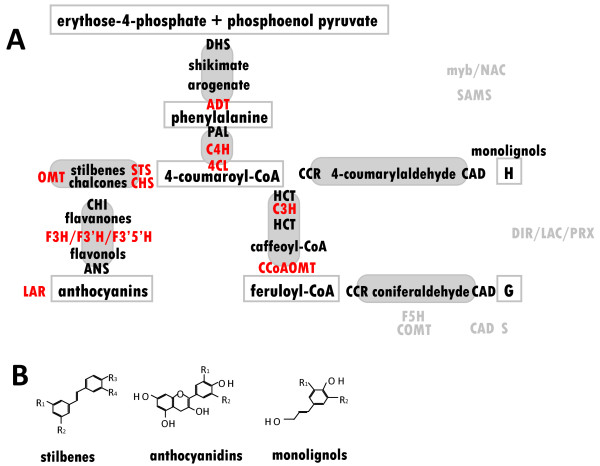
**Schematic overview of the phenylpropanoid pathway**. A Gene families of the main branches for which spruce gene members were profiled by microarray analysis are indicated in red. Given in grey are transcription factors, S-adenosylmethionine synthetase and gene families not part of the core phenylpropanoid pathway in gymnosperms; B examples of metabolites representing the structural variety generated within the core phenylpropanoid pathway, stilbenes: R1-R2-R3-R4: (OH, OCH_3_)-(OH, O-glucopyranose, OCH_3_)- (OH, OCH_3_, H)-(H, OH), [105]; anthocyanidins: R1: (OH, H), R2: H, [106]; monolignols: R1-R2: H-H (p-coumarylalcohol), R1-R2: H_3_CO-H (coniferyl alcohol), R1-R2: H_3_CO- H_3_CO (sinapyl alcohol), [10].

The importance of specialized phenolic compounds for plant chemical defense has been the subject of intensive study in angiosperms ([11,12]), however, knowledge in gymnosperm lineages is sparse. In general, phenolics participate in pre-formed defenses (toxins, cell wall associated compounds) as well as in active defense reactions (antimicrobial phytoalexins). Biosynthesis occurs through a limited number of metabolic pathways (Figure 1A), however, the extreme structural diversity of compounds found so far (Figure 1B) is due to biosynthetic enzymes that belong to multi-member gene families. Phenylpropanoid biosynthesis and its genetic basis has been recently reviewed [13]. For poplar, gene families involved in phenylpropanoid biosynthesis exhibit an expansion and diversification in comparison to short-lived annuals [14], suggesting diversification of chemical defense strategies. The limited number of phenylpropanoid pathway genes functionally characterized in gymnosperm species include those involved in angiosperm lignin biosynthesis (peroxidases: *Picea abies *[15], O-methyltransferases: *Pinus taeda *[16], *P. sylvestris *[17], *P. radiata *[18], hydroxycinnamoyl-CoA:shikimate hydroxycinnamoyltransferase: *P. radiata *[19], and MYB: *P. taeda*: [20]).

Constitutively present phenolics have high priority as defenses against bark-boring insects in long-lived conifers, but pre-formed defenses are regarded costly for the plant since "assimilates" are diverted. This impacts growth rate and reproduction [21]. Our previous findings (unpublished and [22]) already reflected the biochemical link between growth and established (constitutive) defenses in this important metabolic pathway.

Conifers have extremely large genomes (ca. 20 billion bases), which largely consist of repetitive elements ([23,24]), but the enormous size of conifer genomes is also likely due to complex gene families that have been significantly enlarged from angiosperm ancestors. These families have structural and regulatory functions related to defense/resistance mechanisms against pests and pathogens (including terpene synthases, cytochromes P450, TIR-NBS-LRR genes, pathogen-resistance genes and dirigents [25-29]). Those multigenic families resulted from duplications of genes that further diverged.

In the plant kingdom, angiosperms are well studied compared to ancient lineages such as mosses, ferns, or gymnosperms [30]. Therefore, we must rely upon known and characterized candidates from angiosperms to gain insight into the evolution of sequence orthologs of conifer phenylpropanoid-like genes. This involves evidence for neofunctionalization, subfunctionalization or neo-subfunctionalization within gene families. Gene duplication is recognized as an important mechanism for adaptive evolution in plants [31]. Duplication allows "neofunctionalization" or the retention of ancestral function by one gene and the origin of new functions by the second gene. Likely, the novel gene function adopted by one copy of the duplicated genes, which now determines their functional differences, is co-opted from a secondary ancestral property already present before the duplication event [32]. The fate of specialized terpene synthases in spruce is one well-studied example that exemplifies how neo-functionalizations account for functional plasticity in a multi-gene family [33].

Another mechanism for maintenance of gene duplicates involves gene conservation using gene-dosage effects and functional redundancy [34]. Moreover, loss of genes and regulation of gene activity through transcript accumulation are other important aspects to consider in gene evolution. Phylogenetic trees are robust for prediction of orthologous genes in the presence of gene losses and varying rates of evolution among the sampled taxa [35]. Hence, in our study of multi-gene families in spruce we employed phylogenetic reconstruction to facilitate gene annotations.

In the present work, we determined the extent of gene co-expression by correlation analysis for 428 EST microarray elements (Additional File 1). The analysis included a number of multi-gene families originating from 26 biosynthetic genes, three structural genes related to the phenylpropanoid pathway, transcription factors NAC and myb (of which few have been characterized as key regulators of secondary wall biosynthesis and phenylpropanoid metabolism [36,37]), and SAMS (a key enzyme for methylation reactions which are also important in lignin formation [38]) (Additional File 1).

The same ESTs were studied by means of "genetical genomics" to investigate the genetics of defense mechanisms of white spruce against the white pine weevil (*Pissodes strobi*). Genetical genomics [39] combines gene expression with genotyping to map quantitative trait loci (QTL) for gene expression. This new approach to the study of quantitative genetics allows rapid and direct discovery of genes underlying a trait of interest ("positional candidate genes") or genes closely regulated with the QTL by "genetic co-localization" (co-segregation of transcript variation of genes with the phenotypic trait of interest). For example, due to the genetic as well as environmental and developmental sources of variation, transcript level variation at a gene locus underlying a QTL is highly associated with the total phenotypic variation in the actual quantitative trait [38]. Hence, in systems where the environment shows a strong effect on the phenotype, this approach has advantages over conventional QTL analysis with low resolution that further necessitates positional gene cloning [38].

## Results

### Genetical Genomics Reveals Candidate Genes for Weevil Resistance and Genomic Regions with Regulatory Hotspots

In the present study we merged genetics and transcriptomics [40,41] to better understand weevil resistance in spruce, an important phenotypic trait that defines the life history of this economically valuable perennial plant organism. Gene expression variation represents the phenotype most directly related to DNA sequence polymorphism, such that in principle each transcript has a corresponding gene with known position in the genome [42]. Genetical genomics allows assaying thousands of these gene expression traits simultaneously and thus provides data on a large and unbiased set of traits (*ibidem*); these 'expression phenotypes' are then accessible to standard QTL analysis. Using the gene expression traits obtained from our transcriptome study we achieved fine scale phenotyping that was merged with phenotyping of the conventional traits. This allowed us to identify positional candidate genes for any phenotypic variation by testing for co-segregation of markers with gene expression, individual resistance traits, or the composite trait represented by all sampled defense traits (see below). This way, we found certain members of complex gene families within the phenylpropanoid metabolic pathway individually associated with weevil resistance.

**Figure 2:**
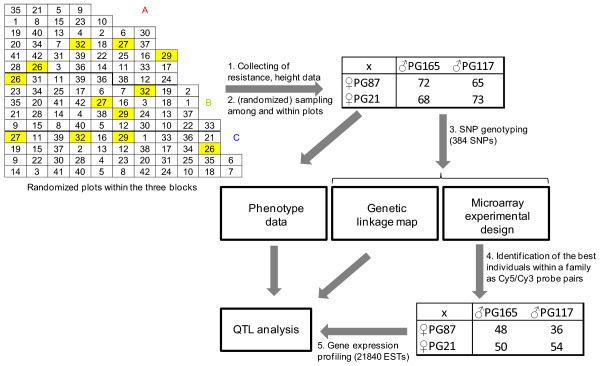
**Outline of the experimental setup for QTL identification**. Layout of study site showing the randomized location of plots (QTL mapping families PG87*PG165 (cross 26), PG87*PG117 (cross 27), PG21*PG165 (cross29) and PG21*PG117 (cross32), in yellow) within blocks A, B, C. The numbers of individuals per family for genotyping, phenotyping and microarray analysis, respectively, are given. For details about which individuals were directly compared using the distant pair design, see Additional File 5.

We used the experimental setup as presented in Figure 2. Plant material was harvested - as outlined in the Methods section - from the progeny of resistant-female-by-susceptible-male crosses, which showed wide segregation for weevil resistance and had shared parentage ([43]). Thus, cross 26 of ♀PG87*♂PG165, cross 27 of ♀PG87*♂PG117, cross 29 of ♀PG21*♂PG165 and cross 32 of ♀PG21*♂PG117 forming the partial diallel were chosen for further analysis. The plant material used for expression profiling (bark tissue from tree leaders) was harvested in a randomized fashion to minimize bias due to the sampling procedure. DNA from an expanded mapping population (417 individuals in total) was genotyped using a custom-built 384 multiplexed SNP chip. This information was used to estimate pairwise recombination rates between SNP loci and subsequently construct the framework genetic linkage map for localizing the QTLs (Additional Files 2 and 3). Phenotypic information for QTL analysis was obtained from (a) tree height, (b) weevil attack, (c) oviposition, and (d) transcriptomics data. Measures were taken for the initial tree height in 1995, and heights in years three and five as well as leader length in year five preceding the artificial augmentation of the local weevil population in October of the same year. Attack rates in 2000 and 2001 were classified as successful top kills, failure to kill the leader, and no attack. For the same years, egg counts along the leaders were summarized into five discrete classes. Comprehensive information about these measures can be found in Additional File 4. In the transcriptomics experiment a two color 21.8 k spruce EST array was employed for gene expression profiling in the partial diallel progeny. A distant pair design that maximized direct comparisons between different alleles at each locus [41] was modified for outbred individuals (Additional File 5). Signal intensities can be found under the GEO accession number GSE22116. We used the signal intensity at each EST spot for the following analyses: (a) generation of expression QTLs (eQTLs) and (b) establishing a co-expression network. In the latter the gene-gene interactions were assessed. Specifically, a Gaussian model and a shrinkage method were employed to evaluate direct gene connections (see Methods). QTLs were mapped in the diallel progeny using a likelihood function to assess the phenotype effect conditional on genotypic variation. A QTL was significant at LOD≥3.84 (Additional Files 6 and 7). A goodness-of-fit test assuming a uniform distribution was performed to test whether the observed frequencies of eQTLs along the linkage map differed significantly from the expected value. Following the rejection of this null hypothesis "eQTL hotspots" signified eQTL clusters with ≥38 eQTLs at a given locus. Positional candidate genes were identified by collocation of at least 40% of their eQTLs with phenotypic trait QTLs based on the criteria for identifying significant QTLs (10,000 randomizations, p ≤ 0.05). The positional candidate genes for the general 'resistance' trait were identified based on strong collocations of gene expression variation with all six studied resistance traits (see Material and Methods).

**Figure 3:**
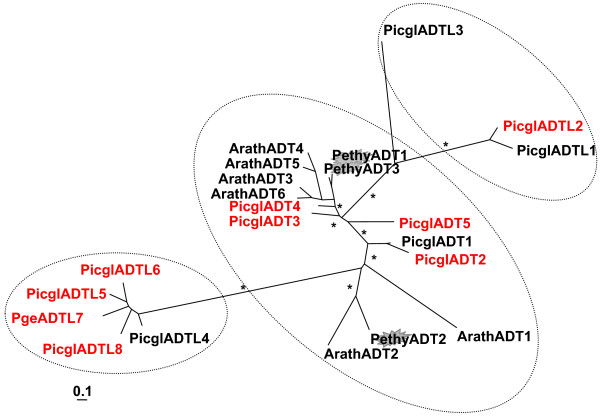
**Arogenate dehydratase (ADT) and related families**. The flash highlights functionally characterized ADT. The bar represents 0.1 amino acid changes. Asterisks indicate 80% and above confidence through bootstrap values. The dashed circles indicate the distinct core clade of the *bona fide *ADT and two related spruce ADT-like (ADTL) families. Arath, *Arabidopsis thaliana*; Picgl, white spruce (*Picea glauca*); Pge, interior spruce (*Picea glauca x engelmannii*); Pethy, petunia (*Petunia x hybrida*); the red font indicates representation on the microarray. GenBank accession numbers are given in Additional File 14.

**Figure 4:**
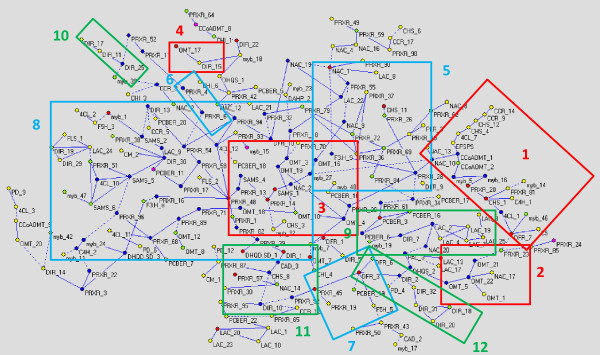
**Co-expression network for the phenylpropanoid pathway**. See also Additional File 1 for annotations (probability cutoff for "true connections" between gene pairs was 0.80; connections are not drawn to scale), vertices marked in the network: red: positional candidates for resistance trait, green: for growth trait, magenta: for both resistance and growth trait (see text); wide range subgraphs can be followed by blue vertices, important connections are framed and are numbered by order of appearance in the Results and in the Discussion, red: focus on O-methyltransferases, blue: peroxidases; green: dirigent-likes.

Expression variation from 428 gene spots generated 4,221 significant eQTLs (LOD ≥ 3.84). Figure 1A shows the phenylpropanoid pathway, gene families from the core branch as well as related families; data relevant for this study are found in Additional File 1. Phenylpropanoid pathway-linked genes that showed strong association with the general 'resistance' phenotype were: SAMS, one ADT-like gene (PicglADTL8, Figure 3), an annotated spruce CYP750 (C24, Additional File 8), and a member with similarity to LAR. An additional 34 phenylpropanoid-related candidate genes were identified based on significant co-segregation with individual weevil resistance traits such as attack rates and oviposition estimates: transcription factors (seven putative mybs and one putative NAC), putative members of the upstream shikimate pathway (DAHP and DHQD-SD), one putative PAL, two annotated CHS as well as one STS, two OMTs from group C and one from group D (OMTL), two P450s (CYP75/F3'H and CYP750, respectively), three different representatives of phenylpropanoid reductases (PCBER, PLR and IFR), one DIR (f-family), six PRXR (including the stress inducible PicabPRX2 orthologue), two putative LACs, one putative catechol-O-methyltransferase and two genes with similarity to LAR. Most associations were found for weevil susceptibility measured in the year 2000 and for both years 2000 and 2001. No dedicated monolignol biosynthetic gene co-segregated extensively with any resistance trait. However, 29 genes from our gene set were associated with individual height growth traits (predominantly tree height measures taken in 1999 preceding the artificially enforced weevil attacks), Additional File 1. As many as 11 peroxidases co-segregated with growth traits, among them several genes which are implied in lignin polymerization by radical coupling of the monolignols. Their co-expression pattern with other phenylpropanoid-related genes is shown in Figure 4.

**Figure 5:**
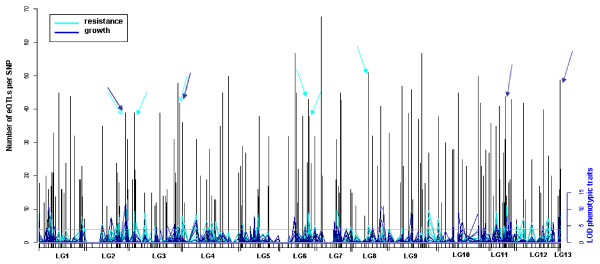
**Representation of the eQTL density map with overlapping positions of pQTLs at individual marker positions**. Linkage groups (LG1-13) are displayed horizontally, black bars indicate SNP marker positions in linkage groups (see also Additional File 2); arrows mark positions with at least three pQTLs (LOD > 3.84, i.e., values above horizontal line) and eQTL numbers ≥ 38.

We superimposed at a given SNP marker pQTL maps of individual phenotypic traits and counts of significant eQTLs from the studied gene set (Figure 5). We found regulatory hotspots comprising multiple pQTLs and accumulated eQTLs. Along this QTL density map, eight loci represented hubs of *trans*-eQTLs, which also corresponded with at least three pQTLs: four loci were associated exclusively with resistance QTLs, and each two loci with growth QTLs and QTLs from individual traits of both growth and resistance, respectively (Figure 5). The composition of those eQTL hotspots with extensive pQTL overlap is given in Additional File 9.

**Figure 6:**
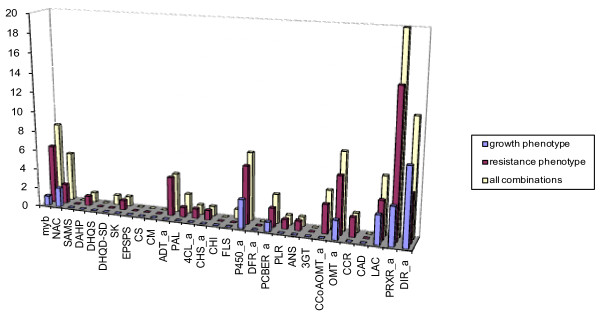
**Representation of 29 studied gene families at eight pQTL associated eQTL hotspots**. Gene families are presented in the following order: transcription factors, early shikimate pathway, the core, and the late phenylpropanoid pathway. Vertical bars show numbers of members of gene families with members having eQTLs at a minimum of two different phenotype-associated eQTL hotspots (all combinations), eQTLs among pure resistance hotspot(s) and other hotspots with resistance association (resistance phenotype associated) as well as eQTLs among pure growth hotspot(s) and other hotspots with growth association (growth phenotype associated), see also Additional Files 9 and 10; "a"... spruce gene family with known/determined phylogeny.

Since the phenylpropanoid pathway proved to be important in insect resistance, we looked at the representation of individual branches within the pathway as well as individual gene families among those regulatory hotspots that were associated with pQTLs. We have summarized the average number of detected eQTLs per gene family as well as gene families whose members have eQTLs at a minimum of two regulatory hotspots with pQTL association (Additional File 10 and Figure 6). Specifically, we compared members from transcription factors myb and NAC, the shikimate pathway, PAL, C4H and 4CL (main branch of the phenylpropanoid pathway), and gene families that are involved in the biosynthesis of various secondary compounds as well as lignin and lignans, respectively (Additional File 10). Among all pathway branches, the branch of the phenylpropanoid pathway that is directly involved in the biosynthesis of the secondary compounds generated on average the highest number of eQTLs, whereas the shikimate pathway generated the lowest number of eQTLs on average. Also, the transcription factors followed by the pathway branch that is directly related to lignin/lignan biosynthesis contributed on average the most members to resistance associated hotspots (Additional File 10). In 20 out of 29 studied gene families, individual family members contributed eQTLs to at least two different phenotype-associated eQTL hotspots. For example, members of *CCoAOMT*, *CCR*, *ADT*, as well as *myb*, *PRXR*, *OMT, P450 *families had eQTLs preferentially associated with resistance hotspots (Figure 6). Specifically, we found ADTs (and one ADT-like gene), OMTs from group C as well as both AEOMT genes, the lignin-forming genes PiglCCoAOMT3, PicabPRX1 and PicabPRX18 orthologs and the stress inducible PicabPIPRX gene, and the P450s that are involved in a wide range of derivatization reactions (CYP75/F3'H, CYP750, F3'5'H/F3H and C4H class, respectively), Additional File 9. The *DIR *gene family members had eQTLs predominantly associated with growth trait QTLs (Figure 6) that were mainly representatives of the f-family (Additional File 9).

Although most spruce gene markers used to build our framework linkage map lack consistent annotations (67% using TAIR7, 54% using Viridiplantae databases gave no hits), Additional File 2, for the few loci that are potential gene expression regulators located within eQTL hotspots and affect highly complex phenotypes, we further infer their putative gene function in spruce. For example, the growth trait associated locus on LG13 with accumulated eQTLs from 49 array elements (Figure 5) represents a glutamate decarboxylase (GAD) gene. GAD catalyzes gamma-aminobutyric acid (GABA) synthesis via decarboxylation from the amino acid glutamate. The regulation of GAD enzyme activity is vital for normal plant development, and is accomplished by calcium/calmodulin binding to the specific CaM domain of GAD allowing the plant to respond to various external stimuli [44]. Recently, the locally enhanced production of GABA in the plant was shown to be connected with a deterrence reaction of the host responding to herbivore attack [45]. Two loci that encode *bona fide *CCoAOMT genes (CCoAOMT-1, CCoAOMT-2) on LG6 represented eQTL hotspots that overlapped with QTL regions for various resistance traits (Figure 5). At the two different CCoAOMT loci a common set of pQTLs (i.e., atk_2000, egg_2000 and sum_egg) as well as eQTLs (generated by 24 members of 11 different gene families) clustered. Most prominently the following gene families were involved: *PRXR *(PicabPRX18, e.g.), *DIR *(PgeDIR13, a-family; PicsiDIR31, PicsiDIR27, f-family), *OMT *(AEOMTs PicglOMT-17 and PicglOMT-18; PgOMT-35, clade II), *myb *(TT2 orthologue, e.g.) and *ADT *(PicglADT3, PicglADT4), Additional File 9. Both *CCoAOMT *genes have also *cis*-eQTLs (Additional File 9) that likely represent promoter polymorphisms involved in the differential expression of the genes. Our finding of *cis*-eQTLs connected by extensive *trans*-regulatory interactions describes an example of epistasis that is commonly observed in the regulation of pathway biosynthetic genes [46].

### Arogenate Dehydratase and Related Sequences

While arogenate dehydratase activity was first detected in *Nicotiana sylvestris *[47], gene cloning and functional characterization was only recently reported in *Petunia hybrida *[48]. Five putative white spruce arogenate dehydratases (ADT) were identified with similarity to characterized bacterial and fungal prephenate dehydratase (PD). They group together with *A. thaliana *and *P. hybrida *(Figure 3) implied in the catalysis of the last step of the shikimate pathway specific for the biosynthesis of phenylalanine (the entry molecule for the core phenylpropanoid pathway) (Figure 1). In addition, eight ADT-like white spruce genes fall into two more remote clusters of the small family (Figure 3).

Four ADT genes as well as six ADT-like genes were represented as elements on our microarray. The ADT-like gene PicglADTL8 was identified as positional candidate for the general resistance phenotype *per se*, Additional File 1. In the co-expression network three ADTs (PicglADT2 WS00810_A15, PicglADT3 WS00921_B18, and PicglADT4 WS0261_E23) as well as one ADT-like (PicglADTL7 WS00937_M15) were present ("PD", PD_4, PD_6, PD_12 and PD_9, respectively; Figure 4). Despite the central importance of ADT in generating the precursor molecule phenylalanine, ADTs were positioned at the edges of long-range subgraphs in gene-gene interactions, Figure 4 (8), (12). PicglADT3 was significantly co-expressed with a class II 4CL gene WS01010_M10 (4CL_11), see below (Figure 4 (8), supporting its function as ADT. Expression of PicglADT2 is negatively correlated with the cluster of constitutive dirigents of the f and b/d class (DIR_2, DIR_32, DIR_31, DIR_20, DIR_18), Figure 4 (12). The ADT-like PicglADTL7 was co-expressed with OPCL PicglACL10 (Figure 4, 4CL_3 WS00729_F23) suggesting both genes act in a related pathway.

### 4-Coumarate:CoA-Ligase (4CL) and Related Acyl CoA Ligases (ACL)

**Figure 7:**
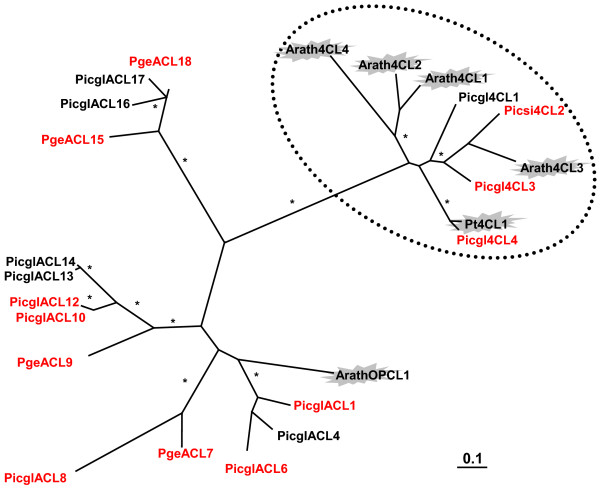
**Acyl-CoA ligase family with 4-coumarate:CoA ligases (4CL) and oxo-pentenyl-cyclopentane (OPC) ligases, and related acyl-CoA ligases (ACL)**. The flash highlights functionally characterized polyketide synthases. The bar represents 0.1 amino acid changes. Asterisks indicate 80% and above confidence through bootstrap values. The dashed circle indicates the distinct clade of the *bona fide *4CL. Arath, *Arabidopsis thaliana*; Picgl, white spruce (*Picea glauca*); Pge interior spruce (*Picea glauca x engelmanii*); Picsi, Sitka spruce (*P. sitchensis*); Pinta, loblolly pine (*Pinus taeda*); the red font indicates representation on the microarray. GenBank accession numbers are given in Additional File 14.

In the biosynthesis of lignin, soluble and wall bound phenolics and flavonids, 4-coumarate:CoA ligase (4CL; EC 6.2.1.12) plays a gate keeper role as the enzyme generates not only the CoA ester of coumaric acid in the last step of the core phenylpropanoid pathway (Figure 1), but the higher substituted coumarate derivatives, such as caffeic acid, ferulic acid and 5-hydroxyferulic acid, and sinapic acid in some angiosperms as well. 4CL is part of a family of adenylate-forming enzymes present in all organisms, including an Arabidopsis gene recently shown to encode a functional 3-oxo-2(2'-[Z]-pentenyl)-cyclopentane-1-octanoic acid (OPC-8) CoA ligase (OPCL) catalyzing an essential step in jasmonic acid biosynthesis [49,50]. The 4CL gene family is moderately sized [14] and shows four *bona fide *4CL genes for spruce (Figure 7). Three white spruce 4CL candidates clustered closely with Arabidopsis 4CL3 (involved in flavonoid biosynthesis) and one spruce gene was found orthologous to loblolly pine 4CL1 (for which a role in the biosynthesis of lignin in compression wood was implied) (Figure 7) [51,52]. In the context of adenylate-forming acyl-CoA ligases, 11 white spruce candidates fell into four divergent clusters, out of which four closely grouped with the Arabidopsis OPCL, indicating a similar function (Figure 7). While this represents a large subfamily, our knowledge about the function of the members is limited.

Of 13 array elements with similarity to acyl-CoA ligase genes, three represent sequence orthologs of *bona fide *4CL genes, while ten elements have higher similarity to oxo-pentenyl-cyclopentane (OPCL), Additional File 1 and Figure 7. Two spruce 4CL genes (Picgl4CL3 WS00112_J15 and Picsi4CL2 WS01010_M10) were present in our co-expression network, Figure 4. They represent spruce classII 4CL genes, which are involved in the formation of flavonoids and soluble phenolics. Picgl4CL3 (4CL_1) was directly co-expressed with two positional candidates for resistance traits (Picsi-PKS22 WS0014_M21 and LAR WS00929_C24), Figure 4 (1). Picsi4CL2 (4CL_11) is co-expressed with PicglADT3, Figure 4 (8). The absence of the classI representative Picgl4CL4 (Additional File 1, Figure 7) and other lignin forming genes (CCoAOMT, and laccases implicated in constitutive lignifications [53]) along with the presence of other gene family members such as those involved in defense mechanisms like dirigents (a-family), P450s, polyketide synthases and phenylpropanoid reductases (see also Additional File 1) suggests higher importance of the recovered gene-gene interactions for defense mechanisms than for normal plant development.

### CCoAOMT Superfamily, Related to Caffeoyl-CoA O-Methyltransferase

**Figure 8:**
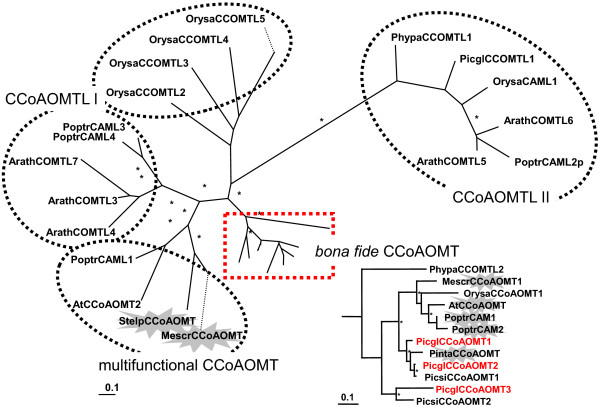
**The caffeoyl-CoA O-methyltransferase family**. Unrooted maximum likelihood phylogenetic tree. Highlighted are clusters of the multifunctional CCoAOMT, the subfamily of CCoAOMT-likes I and II, a rice specific subfamily without dicotyledon representatives and in the inset the expanded *bona fide *CCoAOMT; Picgl, white spruce (*P. glauca*); Picsi, Sitka spruce (*P. sitchensis*); Pinta, loblolly pine (*Pinus taeda*); Arath, *Arabidopsis thaliana*; Poptr, poplar (*Populus tremuloides*); Orysa, rice (*Oryza sativa*); Phypa, *Physcomitrella patens*; Mescr, ice plant (*Mesembryanthemum crystallinum*); Stelp, *Stellaria longipes*. The scale bar represents 0.1 amino acid substitutions per site; asterisks indicate clades with bootstrap confidence greater than 80% and the flash highlights functionally characterized genes. The red font indicates representation on the microarray. GenBank accession numbers are given in Additional File 14.

Caffeoyl-CoA *O*-methyltransferase (CCoAOMT, EC 2.1.1.104) catalyses O-methylation of the hydroxyl group at the C3 position of the phenolic ring in conversion of caffeoyl-CoA to feruloyl-CoA (Figure 1) and has been characterized in many plants, including loblolly pine, where it is associated with developmental lignification [54]. Three white spruce genes are closely related with a loblolly pine CCoAOMT in the clade of *bona fide *CCoAOMT (Figure 8). The diversity observed in the white spruce CCoAOMT clade is the result of lineage-specific gene duplications that were retained throughout evolution.

All three genes involved in monolignol biosynthesis were absent from the co-expression network. However, PicglOMT1, the spruce specific remote OMT (CCOMTL) (Additional File 11) that is putatively involved in phenylpropanoid metabolism and a positional candidate for Ht_1997 growth trait (Additional File 1), was represented by two array spots (IS0014_O19, WS0022_P17) in the network. These two genes termed CCoAOMT_1 and CCoAOMT_2 were directly connected to transcription factors (NAC_10 WS00911_P24 and myb_5 WS00716_G01, respectively) as well as to a putative shikimate EPSPS synthase WS0085_B05, Figure 4 (1). Furthermore, two catechol-O-methyltransferase-like genes WS0107_O19 and WS01013_K15 (Additional File 1) resided at the edges of the network (CCoAOMT_9, CCoAOMT_8, Figure 4): The first clustered with various annotated genes (PicglOMT-13 WS0261_A24, OPCL WS00729_F23, ADT-like WS00937_M15, PgeDIR5 WS00924_E04, see below and Additional File 1), while the second, a positional candidate for the egg_2001 resistance trait, connected to genes with unknown functions. The phylogeny for said catechol-O-methyltransferase (-like) sequences suggests they are ubiquitous genes that lack a counterpart in modern/angiosperm plants (Additional File 12) and are part of a yet to be elucidated pathway in conifer defenses.

### Polyketide Synthases Related to Chalcone and Stilbene Synthases (CHS/STS)

**Figure 9:**
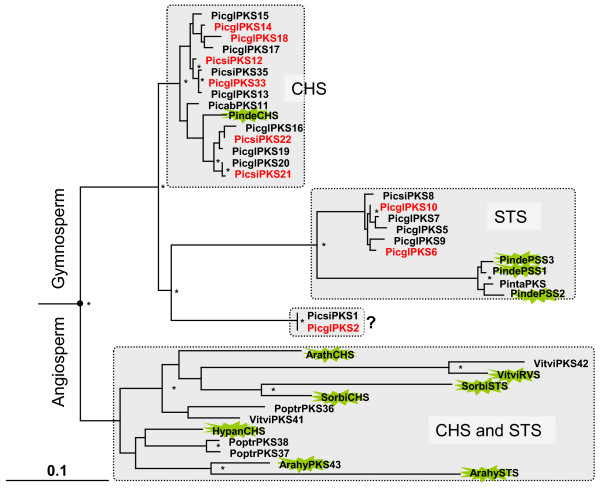
**Phylogeny of higher land plant polyketide synthases with chalcone synthases (CHS), and stilbene synthases (STS)**. Arath, *Arabidopsis thaliana*; Poptr, poplar (*Populus tremuloides*); Vitvi, grape vine (*Vitis vinifera*); Pinde, Japanese red pine (*Pinus densiflora*); Arahy, peanut (*Arachis hypogaea*); Hypan, *Hypericum androsaemum; *Marpo, liverwort (*Marchantia polymorpha*); Sorbi, sorghum (*Sorghum bicolor*); Picab, Norway spruce (*Picea abies*); Picgl, white spruce (*P. glauca*); Picsi, Sitka spruce (*P. sitchensis*); Pinta, Loblolly pine (*Pinus taeda*); The flash highlights functionally characterized polyketide synthases. The bar represents 0.1 amino acid changes. Asterisks indicate 80% and above confidence through bootstrap values and the flash highlights functionally characterized genes. Dashed boxes indicate distinct clades with sub-families. The tree was rooted using the distantly related liverwort stilbenecarboxylate synthase and moss chalcone synthase-like sequences as outgroup (not shown). The red font indicates representation on the microarray. GenBank accession numbers are given in Additional File 14.

Stilbene synthase (STS, EC 2.3.1.95) and chalcone synthase (CHS, EC 2.3.1.74) are plant-specific polyketide synthases at the entry of stilbenoid and flavonoid biosyntheses, respectively (Figure 1). Both types of enzymes perform a sequential condensation of three acetate units to a CoA-ester to form an intermediate that is folded into the aromatic ring systems of naringenin chalcone or the stilbene backbone [55,56]. While flavonoids, which play a vital role as UV protective pigments in plants, are ubiquitous in the land plant kingdom and were reported in the basal lineages of liverworts and mosses [57], stilbenes have so far been detected in relatively few plant families where they contribute to the resistance of woody tissues to degradation and act as phytoalexins. The main constitutive stilbene glycosides in *Picea *species are astringin and isorhapontin [58]. Fourteen spruce CHS-like sequences form a tight cluster with the Japanese red pine CHS, indicating several duplications of functionally related polyketide synthases (Figure 9). While the ancestor of modern polyketide synthases predates the evolution of gymnosperms and angiosperms, the close relationship of STS and CHS within the angiosperms and gymnosperms indicates that these functions evolved independently several times. In gymnosperms distinct clades of both pine and spruce orthologues are found for STS and for CHS, consistent with the presence of both functions in a common ancestor of these lineages.

Strong collocation of gene expression and trait variation identified two CHS genes Picsi-PKS21 and Picsi-PKS22 as well as the STS gene Picgl-PKS6 as potential candidates for weevil resistance traits (sum_egg, egg_2001 and egg_2001, respectively), Additional File 1. Picsi-PKS22 WS0014_M21 is present within the network (CHS_1) and co-expressed with Picgl4CL3 and a peroxidase WS0063_P06 that co-segregated with the sum_atk resistance trait (Figure 4 (1)). Picgl-PKS6 WS00925_G22, (CHS_11, Figure 4 (5)) was negatively co-expressed with peroxidase PicsiPRX2 WS0074_A10 that responded to wounding ([59]). The CHS gene WS0024_K18 (termed CHS_3) from the Picsi-PKS14 cluster is associated with a myb transcription factor (myb_32, Figure 4 (3)).

Picgl-PKS2 WS00731_E22 (see also Figure 9) was negatively co-expressed with a-family dirigent PgeDIR2 WS00911_I09 (Figure 4 (11), CHS_8 and DIR_10). The expression of PgeDIR2 was significantly triggered by insect feeding [29], see below. Interestingly, Picgl-PKS2 was co-expressed with an ANAC057 transcription factor WS00929_E05 (NAC_14, Figure 4 (11)) that accumulated with increasing growth rate (unpublished). The contrasting expression pattern found for diverse members of the polyketide synthase gene family reflects the differential gene regulation for individual members.

### O-Methyltransferase Superfamily, Related to Caffeic Acid/Coniferaldehyde O-Methyltransferase (COMT)

**Figure 10:**
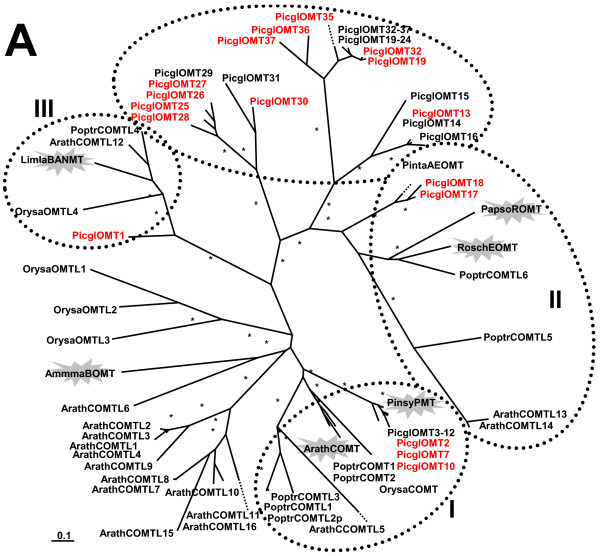
**The O-methyltransferase superfamily family with caffeic acid 3-O-methyltransferase (COMT), pinosylvin O-methyltransferase (PMT), eugenol O-methyltransferase (EOMT), reticulin O-methyltransferase (ROMT), beta alanine N-methyl transferase (BANMT), and bergaptol O-methyltransferase (BOMT)**. A Unrooted maximum likelihood phylogenetic tree; Picgl, white spruce (*P. glauca*); Pinta, loblolly pine (*Pinus taeda*); Arath, Arabidopsis thaliana; Poptr, poplar (*Populus tremuloides*); Orysa, rice (*Oryza sativa*); Ammma, bullwort (*Ammi majus*); Limla, *Limonium latifolium*; Rosch, china rose (*Rosa chinensis*); Pinsy, scots pine (*Pinus sylvestris*); Papso, poppy (*Papaver somniferum*); Highlighted are functionally distinct clusters of COMT/OMT, with (I), (II), and (III) expanded in Figure 11, B; the scale bar represents 0.1 amino acid substitutions per site; asterisks indicate clades with bootstap confidence greater than 80% and the flash highlights functionally characterized genes. The red font indicates representation on the microarray. GenBank accession numbers are given in Additional File 14.

A total 37 of *OMT*/*COMTL *candidates were identified in white spruce through mining of the Treenomix EST database (Spruce V8 database will be published elsewhere, K. Ritland, pers. com.), relative to 17 genes in *Arabidopsis*, eight in poplar, and six in rice (Figure 10). The founding members of the O-methyl transferase superfamily (OMT, EC 2.1.1.6) involved in the synthesis of methylated plant phenolics are related to the formation of the angiosperm monolignol precursor of S-lignin. However, the S-lignin pathway and sinapic acid-derived metabolites, such as syringyl lignins, are absent in gymnosperms (Figure 1). Even though they are broadly represented in the OMT/COMTL superfamily, evidence for dedicated *bona fide COMT *orthologues in white spruce is missing.

**Figure 11:**
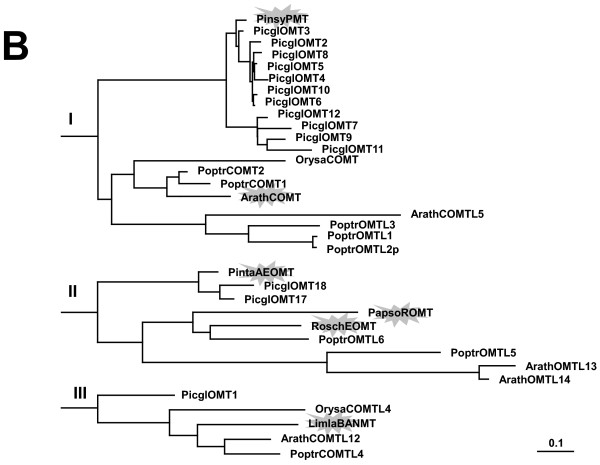
**The O-methyltransferase superfamily family with caffeic acid 3-O-methyltransferase (COMT), pinosylvin O-methyltransferase (PMT), eugenol O-methyltransferase (EOMT), reticulin O-methyltransferase (ROMT), beta alanine N-methyl transferase (BANMT), and bergaptol O-methyltransferase (BOMT)**. B expanded excerpts of functionally distinct clusters (I), (II), and (III) from Figure 10, A; Picgl, white spruce (*P. glauca*); Pinta, loblolly pine (*Pinus taeda*); Arath, Arabidopsis thaliana; Poptr, poplar (*Populus tremuloides*); Orysa, rice (*Oryza sativa*); Ammma, bullwort (*Ammi majus*); Limla, *Limonium latifolium*; Rosch, china rose (*Rosa chinensis*); Pinsy, scots pine (*Pinus sylvestris*); Papso, poppy (*Papaver somniferum*); the scale bar represents 0.1 amino acid substitutions per site; the flash highlights functionally characterized genes. GenBank accession numbers are given in Additional File 14.

OMTs were among phenylpropanoid gene families most completely represented in the co-expression network (Figure 4). Three COMTLs (PicglOMT-11 WS00715_G04/WS0261_F17, PicglOMT-10 WS0023_M11, PicglOMT-7 WS02611_F21) similar to the stress inducible O-methyltransferase from scots pine ([17]; clade I, Figure 10 and 11) were highly co-expressed, OMT_5, OMT_21, OMT_22, OMT_1, Figure 4 (2). Their expression was negatively correlated with the expression of a sequence (WS00716_K11) that shares similarity to AtPER16/AtPER45 and is a positional candidate for the Hgt1999 trait (Additional File 1). Five spruce OMTs (WS0071_H13, WS0078_K09, WS0104_J07, WS0046_C03, and WS01013_K11) that are part of the lineage specific clade in the plant OMT/COMTL superfamily (Figure 10) cluster within the network (OMT_4, OMT_10, OMT_19, OMT_3, and OMT_18, Figure 4 (3)) and were co-expressed with a transcription factor that weakly resembles the secondary wall-associated AtMYB83 (WS0079_B12). PicglOMT-1 WS0099_A18, with strong similarity to a beta-alanine betaine synthase (clade III, Figure 10 and 11), represents a positional candidate for the egg_2000 trait and is co-expressed with PgeDIR5, a dirigent of the a-subfamily, DIR_15, Figure 4 (4). Although their classification here is based on phylogenetic analysis alone, and biochemical characterization needs to support the annotation, spruce genes of the OMT/COMTL superfamily possibly extent the repertoire of spruce defense mechanisms.

### Cytochrome P450s Involved in Oxygenation of Phenylpropanoids

In addition to a major plant carbon sink - the lignin biosynthesis - cytochromes P450 contribute to the biosynthesis of many bioactive phenolic derivatives (for a review see [60]). In the families of the cinnamte hydroxylase (C4H, CYP73) and coumaroyl-shikimate 3'-hydroxylase (C3'H, CYP98) two and one white spruce candidate were identified, corresponding to single genes in Arabidopsis, while no white spruce coniferylaldehyde 5-hydroxylase (CA5H, CYP84) sequence orthologues were found. In the CYP75 family, seven diverse white spruce candidates form two distinct clusters with the Arabidopsis CYP75B1 encoding a functional F3'H [61]. Phylogenetically related, but without biochemical function assigned, 45 white spruce candidates constitute the unusual large and diverse conifer specific CYP750 family, an outgroup to angiosperm CYP84. Another group of P450-dependent monooxygenases forms the spruce-specific outgroup of nine CYP76-likes (Additional File 8).

Within CYP75/F3'H sequences, family member C59 represents a positional candidate for the sum_egg resistance trait. Another representative (C57, WS00931_D17) is present in our co-expression network (Figure 4 (5)) and is negatively correlated with NAC_1 (WS00713_M11), a NAC transcription factor that is a candidate for the atk_2001 resistance trait. The function of the diverse CYP750 family (Additional File 8) is unknown, however, the sequence relationship with CYP84 (F5H), CYP75(F3'5'H/F3'H), CYP98 (C3H), and CYP73 (C4H) which are involved in oxidation reactions of the phenylpropanoid metabolism indicates that these families and CYP750s may share a common progenitor and possibly a structurally similar substrate of the phenylpropanoid class. On our array 11 spruce specific CYP750 genes were represented (Additional File 1), one CYP750 (C24) was identified in our study as a positional candidate for the weevil resistance phenotype *per se*, and three CYP750s (C18, C12 and C56) are found in the co-expression network. C18 (F5H_5, WS00934_G23) was co-expressed with a candidate (LAR similarity, DFR_3) for the general resistance phenotype (Figure 4 (7)). C12 (F5H_3, WS00923_E07) was negatively correlated with myb myb_1 (WS00917_H19) that is loosely related to TT2, a putative regulator of proanthocyanidin biosynthesis [62], and in our study a positional candidate for both atk_2000 and sum_atk resistance traits (Additional File 1, Figure 4 (8)). Expression patterns for both CYP750s suggest diverged, though unknown, functions consistent with their position in different subclades (Additional File 8). The third CYP750 C56 (F3'H_8, WS00922_H05) was negatively correlated to a cluster of co-expressed peroxidase genes of which some are implied in monolignol polymerization (PicabPRX18 IS0011_F24 (PRXR_1), spruce ArathPER42 WS0031_G05 (PRXR_13), and ArathPER64 WS01016_D12 (PRXR_58) orthologues, see elsewhere) (Figure 4 (8)). Future characterization of spruce CYP750 candidates will need to uncover their enzymatic function *in planta*.

### Phenylpropanoid Reductases

**Figure 12:**
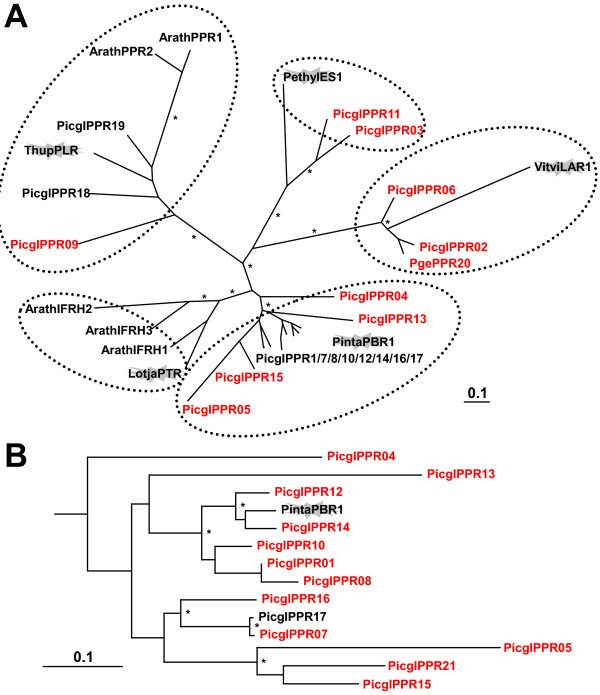
**The phenylpropanoid reductase family with leucoanthocyanidin reductase (LAR), pinoresinol-lariciresinol reductase (PLR), phenylcoumaran benzylic ether reductase (PBR), and isoeugenol synthase (IES)**. A Unrooted maximum likelyhood phylogenetic tree (isoflavonone reductase homolog, IFRH); B Expanded conifer phenylcoumaran benzylic ether reductase family, rooted with *Lotus japonicus *pterocarpan reductase. Thupl, western red cedar (*Thuja plicata*); Lotja, *Lotus japonicus*; Pinta, loblolly pine (*Pinus taeda*); Vitvi, grape vine (*Vitis vinifera*); Arath, *Arabidopsis thaliana*; Picgl, white spruce (*Picea glauca*); Pge interior spruce (*Picea glauca x engelmanii*); the flash highlights functionally characterized genes; asterisks indicate 80% and higher bootstrap values; the scale represents 0.1 amino acid changes. The red font indicates representation on the microarray. GenBank accession numbers are given in Additional File 14.

Phenylpropanoid reductases are a class of closely related enzymes that include leucoanthocyanidin and isoflavone reductases (LAR/IFR), pinoresinol and lariciresinol reductasess (PLR), phenylcoumaran benzylic ether reductase (PBR), and isoeugenol synthase (IES) that have been shown to participate in the biosynthesis of a plethora of constitutive and induced defense related phenylpropanoids and phytoalexins, such as lignans and isoflavans [63-67]. With the exception of the IFR family, for which no white spruce homologues were identified, at least two white spruce candidates were detected in the other families (Figure 12A). A total of 12 white spruce homologs cluster closely with the loblolly pine PBR1 (Figure 12B) which reduces the benzylic ether functionalities of both dehydrodiconiferyl alcohol and dihydrodehydrodiconiferyl alcohol in the formation of 8-5'-linked lignans [66]. However, the phylogenetic distance of the white spruce candidates to the functionally characterized angiosperm and gymnosperm phenylpropanoid reductases does not allow functional predictions extending beyond similarity of the mechanism of the catalyzed reaction.

Out of 21 array elements annotated as phenylpropanoid reductases 11 were present within the co-expression network ("PCBER", Figure 4). Gene expression variation of PicglPPR05 (represented by three array elements: WS00723_J06, WS0048_K01, WS00920_D17 (Figure 4 (9)): PCBER_6, PCBER_3, PCBER_16) was co-expressed with the secondary wall-associated AtMYB20 (myb_19; WS0071_H14), which was a candidate for the ldr99 growth trait. PicglPPR21 is a positional candidate for the sum_egg resistance trait; its transcript abundance was lowest in the spruce mapping family with the most vigorous growth and highest weevil attack rate (unpublished). In the network this PCBER gene (WS0086_D12; PCBER_11) was connected to PicglDIR10 (WS0261_J16; DIR_30), a member of the b/d-subfamily and the cluster of peroxidases involving monolignol forming enzymes (AtPER64, AtPER42, PicabPRX18, see above, and PopalPRX WS0099_B17 (PRXR_51)), Figure 4 (8). Transcripts of PicglPPR13 (WS01011_J14) accumulated with increasing growth rate, and PicglPPR13 (PCBER_19) was negatively co-expressed with the resistance phenotype candidate LAR WS00725_B17, DFR_3, Figure 4 (7). The latter is correlated with another (remote) LAR representative WS00926_B24 (DFR_6) that was lowest expressed in the spruce mapping family 26, the stress inducible PicabPRX2 WS00928_G19 (PRXR_45), and the cluster of dirigents involved in constitutive defenses, DIR_2, DIR_32, DIR_31, DIR_18, DIR_20, Figure 4 (12), see also below. The expression pattern for PicglPPR13 suggests that this PCBER could be involved either in susceptibility or tolerance to insect feeding. However, for other representatives (PicglPPR21) their co-expression pattern suggests involvement in constitutive defenses.

### Dirigent Proteins

A recent study on Sitka spruce dirigent-like proteins (DIR) [29] reported 35 unique DIR or DIR-like genes. For our survey, we were able to use expression data from 23 elements annotated as unique DIRs and represented on the microarray covering distinct a-, b/d-, and f-subfamilies (Additional File 1). The DIR f-subfamily is spruce specific, while angiosperm members can be found for both a- and b/d- subfamilies, [29] showed that DIRs from different clades have distinct gene expression patterns with a suggested role in induced defense to weevil feeding for members of the a- subfamily. Representatives of b/d- and f-subfamilies are more likely involved in primary processes or constitutive defense (*ibidem*).

Within the network PicglDIR12 WS00815_A07 (b/d-subfamily, DIR_7) was co-expressed with a growth trait associated transcription factor resembling secondary wall-associated AtMYB20 (myb_19) and with a cluster of six putative laccases, Figure 4 (9). A cluster of constitutive dirigents involving b/d-subfamily members (PicsiDIR17 WS01012_J06, PicsiDIR11 WS01012_K18, PicsiDIR21 WS02610_M19, PicglDIR7 WS0262_G08 (Additional File 1)), and PicsiDIR26 WS0058_H22 from f-subfamily were co-expressed with remote LAR (DFR_3), one of the identified positional candidates for weevil resistance, Figure 4 (12).

PgeDIR13 (WS00914_H24) was highly up-regulated in bark tissue following weevil feeding and for PgeDIR2 (WS00911_I09), another a-subfamily member, the highest induction was registered, [29]. In the network, PgeDIR13 (DIR_11) was co-expressed with two other a-subfamily members (PicsiDIR19 WS01011_J07, PicsiDIR16 WS01032_M02), DIR_17, DIR_25, Figure 4 (10), and for PgeDIR2 gene expression was negatively regulated with the expression of the polyketide synthase Picgl-PKS2, CHS_8, Figure 4 (11). Thus, our experiment confirms previous observations that dirigent subfamilies in constitutive defenses are differentially regulated than dirigents in induced defenses. Similar to the cluster of spruce/conifer-specific members from the OMT superfamily (OMTL), see above, dirigents from the same subfamily are also strongly co-expressed. This provides evidence for the presence of a multitude of gene copies with the same or highly similar protein functions that act in related pathways.

### Class III Peroxidases

Plant class III peroxidases represent a key multifunctional enzyme family that is involved in such diverse processes as auxin catabolism, defenses, general stress responses, and lignification [68]. Peroxidases were suggested to function in the radical reaction of monolignols to the polymer lignin [69]. Misregulation of peroxidases provided indications for altered lignin composition ([70], review of xylem class III peroxidases in lignification: [71] and [72]). Recently, two lignin-forming peroxidases have been identified and characterized from Norway spruce suspension culture showing substrate preference for H- or G-lignin precursor molecules, respectively [15]. To date, 17 peroxidase unigenes for conifer lignin polymerization have been identified and their expression abundance in different tissues and after biotic and abiotic challenges studied [73].

For eight genes (PicabPRX1, PicabPRX2, PicabPRX3, PicabPRX4, PicabPRX6/PicabPRX7 cluster, PicabPRX16/17, PicabPRX18, PicabPIPRX) the respective white spruce orthologues were represented on the microarray (Additional File 1, Additional File 13). While some peroxidases with proposed roles in lignin polymerization are related to developmental lignification (PicabPRX1, PicabPRX4, PicabPRX16/17 cluster, PicabPRX18, array elements: see Additional File 1), others were stress inducible (PicabPRX2, PicabPRX3, PicabPIPRX, elements: Additional File 1) and reflect the specific stress responses of peroxidases ([73,15]). In addition, gene expression of two other inducible peroxidases (PicsiPRX1 and PicsiPRX2, [59]) were surveyed (Additional File 1). PicsiPRX1 (WS0012_J09, PRXR_4) was co-expressed with several other peroxidases, Figure 4 (6). Constitutive expression levels of PicsiPRX2 (WS0074_A10) increased with higher inherent growth rate of individuals (unpublished). PicsiPRX2 expression (PRXR_26) was negatively correlated with expression of stilbene synthase Picgl-PKS6 (CHS_11), a positional candidate for the egg_2001 resistance trait (Figure 4 (5)). We suggest that PicsiPRX2 and Picgl-PKS6 are involved in different defense strategies. The white spruce PicabPRX2 orthologue WS00928_G19 is significantly associated with the sum_egg resistance trait, and is also co-expressed with the remote leucoanthocyanin reductase, one positional candidate for the "resistance phenotype" *per se*, (PRXR_45, DFR_3, Figure 4 (7), Additional File 1).

The ability to bind lignin was demonstrated *in vitro *for PicabPRX18 [73]. The white spruce orthologue of PicabPRX18 is a positional candidate for Hgt1999 growth trait. PicabPRX16/17 is a positional candidate for Hgt1997 trait. A co-expression cluster involving the white spruce orthologue of PopalPRX WS0099_B17, a putative spruce homologue of the secondary wall-associated transcription factor AtMYB83 WS0051_I02 and S-adenosylmethionine synthetase (SAMS) WS0261_C18 were positional candidates for Hgt_1995 trait (PRXR_51, myb_47, SAMS_6, Figure 4 (8)), Additional File 1. Also, the PicabPRX4 orthologue WS00912_J06 (PRXR_37) was co-expressed with a cluster of peroxidases with growth associations (Figure 4 (5), Additional File 1), among them PicabPRX6/PicabPRX7 (WS00910_L21/WS01025_F04: PRXR_36, PRXR_69). Interestingly, transcript abundance for all genes in this cluster decreased with growth rate. This suggests that a number of peroxidases are important in reinforcement of anatomical structures (via lignification, e.g.). Similarly, lignin biosynthetic genes are down-regulated in fast growing individuals [38].

## Discussion

The stem-borer *Pissodes strobi *damages the host tree by attacking the shoot apical leader of the previous year's growth. The feeding larvae typically consume phloem tissue. In the spruce bark, the secondary phloem and the cambium are very active chemical defense production zones that include secondary metabolites such as the polyphenolics that are abundantly present in the parenchyma. Pre-formed defenses are established co-ordinatedly during the development of secondary xylem in the apical shoot [22].

We studied the complexity of phenylpropanoid-related gene families and gene regulation of individual family members. We found evidence for new pathways in conifer defenses that involve genes without counterparts in modern plants that need to be further investigated. Employing genetical genomics, we showed that this approach is valuable for elucidating interactions between genes that originate from complex families with multiple related sequences. By including weevil resistance and height growth traits in the quantitative analysis, we identified eight genomic regions with extensive accumulation of phenotypic variation from multiple traits coinciding with hotspots of transcript abundance variation. The identified master regulons ranked hierarchically highest among all genotyped loci, since they are likely responsible for massive gene expression variation in the pathway and were linked to changes in the studied phenotypes.

### Phenylpropanoid and Related Gene Families in Spruce

Phenylpropanoids fulfill important functions as defense compounds, e.g. simple hydroxycinnamic acids and monolignols or the structurally complex flavonoids, isoflavonoids, and stilbenes. They have overlapping roles as signaling molecules in plant development and plant defense [74]. The conifer phenylpropanoid pathway is characterized by a higher level of complexity compared to angiosperms ([14] and this study) that might include several not yet resolved alternative pathways. The expansion of phenylpropanoid and related genes into multi-gene families serves either the independent regulation of the biosynthesis of different classes of compounds or the gene dosage through massive accumulation of these compounds [74]. For example, we identified several associations with weevil resistance traits for individual members of the NADPH reductase gene family with a range of product specificity of related phenylpropanoid-derived plant defense compounds (Figure 12). We also identified associations for members within two spruce- (or generally conifer-) specific groups massively represented in the OMT superfamily (Figure 10) and the phenylpropanoid P450s (clade of CYP750, Additional File 8), respectively, both with entirely unknown functions (Additional File 1).

Generally, plant specialized metabolism is characterized by gene duplication events and gene diversifications leading to modified/optimized product specificity and modified tissue specific expression [75]. However, it is currently unclear whether adaptive or non-adaptive forces are more important for the maintenance of gene duplicates [34]. In brief, new pathways were established among different plant species by independent recruitment and inactivation of biosynthetic enzymes through downregulation or loss of certain genes of an ancestral pathway [75]. Interestingly, it has been shown that CCoAOMT-suppression in pine, unlike in angiosperms, generates only moderate reduction in lignin due to compensatory reactions that lead to the incorporation of unusual monolignols (catechyl units) [76]. In the O-methyltransferase family (CCoAOMT sequence similarity) we also found one gene with high similarity to functionally characterized catechol-OMT conserved across species of the eukaryotic and prokaryotic kingdoms, but was lacking in the angiosperms (Additional File 12).

In total, 44 positional candidate genes were directly associated with resistance traits (Additional File 1). Positional candidate genes were prominently identified in expanded gene families such as dirigents and peroxidases, for which some members are implicated in downstream reactions of lignan/lignin formation. Plant class III peroxidases are a key multifunctional enzyme family that is involved in diverse developmental and defense processes ([68,71]). Evidence that certain peroxidases may function in lignin polymerization came from gene misexpression experiments that altered lignin composition [70]. In several gene families of the core lignin pathway, members exist that can function in both stress/elicitor response and developmental lignification [77]. However, given the individual families sizes, phenylpropanoid reductase, CYP750, CHS/STS, and OMT gene families showed the highest proportion of positional candidate genes for resistance. Spruce genes with sequence similarities to COMT (caffeic acid O-methyltransferase) and F5H (ferulate-5-hydroxylase), two enzymes involved in the syringyl (S-) lignin formation in angiosperms, were identified. Due to the lack of these pathways in gymnosperms and to their low sequence similarity, these sequences were annotated as OMTs putatively involved in stilbenoid synthesis and P450s putatively acting in flavonoid synthesis, respectively (see above). It is likely that enzymatic reactions in monolignol/lignin biosynthesis are adopted from conserved ancestral pathways that originally functioned in protection against microbial infection or UV radiation. Interestingly, those reactions emerged in different plant lineages by convergent evolution [78].

### Gene Expression Regulation of Gene Family Members in the Phenylpropanoid Pathway

Microarray experiments that aim to elucidate gene expression differences between treatment and control groups usually involve reference or related designs in a small sample set and cross-hybridizations between gene spots with high nucleotide similarity complicate data interpretation. However, the specific statistical procedure employed in genetical genomics that studies large experimental populations of randomized genetic background reduces this effect. Positions of eQTLs locate genomic regions that harbor regulatory elements that control the expression of a single gene or a subset of genes acting in the same pathway. In the case of *cis*-regulation, the genomic location of the eQTL coincides with the physical location of the regulated gene, while *trans*-acting eQTLs identify regulatory elements for the gene elsewhere in the genome. Distribution of eQTLs may spread evenly on the genome or appear in clusters or in "hotspots" depending on the genetic architecture of gene interactions. In this way, master regulons involved in multiple traits (i.e., pleiotropic genes) can be identified, and the amount of epistasis from interacting loci can be uncovered [39]. We used this approach to identify spruce genes that are potentially important for constitutive defense mechanisms and to establish resistance against *Pissodes strobi*.

Apical leaders of 188 *Picea glauca *individuals were studied for gene expression changes in phenylpropanoid genes prioritized from our 21.8 k spruce EST chip (see Material and Methods for details). Genomic regions with clustered expression and phenotypic trait variation might contain master regulons ([46,79]). Regions underlying *trans-*eQTL hotspots have pleiotropic genetic background (Figure 5). Loci that accumulate pQTLs of both growth and resistance traits may be involved in fitness trade-offs. We identified two such eQTL hotspots on the spruce genome (LG 2 and LG 3). The thorough functional analysis of such largely pleiotropic genes is hampered because their knock-out mutants likely exhibit lethal or highly deleterious phenotypes [46]. However, recently, a locus was characterized which conveys plant resistance to a wide spectrum of pathogens and herbivores and at the same time reduces vegetative growth [80]. The dirigents represent a candidate gene family for which some members were associated with both growth and resistance trait variation, e.g., expression variation for PicsiDIR17 (b/d-family) as well as PicsiDIR31 (f-family) mapped onto eQTL hotspots associated with growth traits and resistance traits (Additional File 9).

Phylogeny and the analysis of the underlying gene expression pattern identified functional divergence among members of gene superfamilies. In several cases gene expression of family members was co-regulated with variation in weevil resistance. eQTLs that accumulated at resistance hotspots were generated from diverged gene family members of *CCoAOMT *(PicglCCoAOMT1, PicglCCoAOMT2, PicglCCoAOMT3, see also below), *OMT *(AEOMT and group C genes), *PRXR *(PicabPIPRX, PicabPRX1, PicabPRX2, PicabPRX18, e.g.), *CYP450 *(CYP75/F3'H, F3'5'H/F3H, CYP750), *DIR *(a- and f-family, e.g.), the *PCBER *(LAR, PCBER), and *4CL *(acyl-CoA ligase, OPCL, 4CL) (Additional File 9). It has recently been shown that tandem gene duplication and subsequent gene retention is particularly common for biotic stress genes [81]. This finding indicates that the increased rate of gene duplication and diversification offers the potential for adaptive evolution and divergence. Thus, the neo-and subfunctionalizations found in the multi-gene families of the spruce phenylpropanoid pathway are possibly highly important for beneficial phenotypic innovations.

Linkage groups on our spruce QTL map were assigned following [82] and the spruce-loblolly pine comparative mapping project (C. Liewlaksaneeyanawin, pers. comm; Additional File 2). Therefore, valuable cross-species comparisons for QTL positions are available for *Pinaceae*, e.g., the two *bona fide CCoAOMT *genes CCoAOMT-1 and CCoAOMT-2 mapped to the spruce LG6 (Additional Files 2 and 9, Figure 5), in synteny with the recently published loblolly pine [83].

Not only did the two *bona fide CCoAOMT *genes harbor eQTL hotspots but also overlapped with QTL regions for the traits atk_2000, egg_2000, and sum_egg (Figure 5). This suggests a close association of monolignol formation with defense against the stem borer *Pissodes strobi*. It is known that cell wall-integral lignin provides a physical barrier for invading pests. Specifically, the lignified parenchyma cells are the important pre-formed anatomical structures that are involved in such constitutive defenses [21]. In addition, localized *de novo *synthesis of lignin with structural similarity to early developmental lignins occurs in response to stress and is associated with wound healing ([84,6,86]). At the two *CCoAOMT *loci, eQTLs were mostly generated from genes of the shikimate pathway, monolignol biosynthesis and downstream condensation reactions, lignan formation, flavonoid biosynthesis, and multifunctional OMT activity. The genomic region harboring the cluster of CCoAOMT genes flags its general importance. We found in our large-scale transcriptomics study that 36% and 53% of the mapped *trans*-eQTLs generated from 1307 and 992 genes, respectively, were commonly shared between both CCoAOMT loci. Certain gene ontology categories were overrepresented and commonly shared between the two eQTL cluster (secondary metabolic process, response to biotic stimulus, e.g.). This suggests interactions between both CCoAOMT loci and other genes that extend beyond the phenylpropanoid metabolism. Further investigations will substantiate this in detail.

In contrast to CCoAOMT-1 and CCoAOMT-2, association with extensive phenotypic variation is currently unknown for CCoAOMT-3. Yet, the three *CCoAOMT *genes represent our most comprehensive example of differential gene regulation for duplicated genes (the phylogeny of the family is given in Figure 8). No *trans*-eQTL from CCoAOMT-1 mapped onto CCoAOMT-2 and vice versa; however, the third *CCoAOMT *gene exhibited *trans*-eQTLs at both loci, suggesting a lower regulatory hierarchy for CCoAOMT-3 among all three genes (Figure 8). CCoAOMT-1 expression variation also mapped onto the growth trait associated eQTL hotspot on LG13, and, generally, the transcript abundance of CCoAOMT-1 was increased with higher inherent growth rate in the experimental population (not shown). CCoAOMT-3 is a positional candidate for hgt1999 trait. At locus CCoAOMT-2 eQTLs from ERFs linked to defensive gene expression (ERFs from group IX [87], specifically, transcription repressors (ERF3, ERF4, ERF7 [88])) accumulated (unpublished results). Consequently, CCoAOMT-2 could indeed function in constitutive resistance. The identified regulators (ERFs) were shown to affect important constitutively expressed defense genes such as basic chitinase and glycosyl hydrolase family 17 protein (beta-1,3-glucanase, [88]). The three monolignol biosynthesis genes were likely retained throughout evolution because of their individual importance and their temporally and spatially dependent recruitment for lignin formation: lignin that is constitutively expressed and related to primary processes, lignin that is wound inducible and participates in active defense, or lignin that is deposited for wound healing.

## Conclusions

This study describes the genetics of pest resistance and utilizes genetical genomics to elucidate the genetic basis and evolution of phenolics based insect resistance in a commercially valuable conifer tree species. The results add to recent QTL studies that have dissected mechanisms underlying the genetic control of wood traits and volume growth in eucalyptus but were completely lacking testing of biotic stress resistance, and in particular, for conifers [89]. We utilized eQTL mapping to conduct a pre-selection of the candidate genes by testing for correlations with resistance traits. The findings can facilitate research efforts on targeted gene association studies specific to nonstructured conifer populations aimed at resolving the genetic basis of host resistance to herbivory.

Our work also provides a substantial account and perspective of the functional characterization of several unresolved alternative pathways in plant defenses. The subfunctionalization of individual gene family members of the phenylpropanoid pathway was evident at the phylogenetic and at the gene expression level. Using genetical genomics, we confirmed that some candidate genes in this pathway were indeed genetically associated with QTLs for white pine weevil resistance. Weevil resistance was also associated with *trans*-regulatory hotspots. At such genomic regions, eQTLs were generated by several subfamilies from multimember families like *4CL, CCoAOMT*, *OMT, CYP450, PPR, DIR*, and *PRXR*. Tandem gene duplication and subsequent gene retention is common for biotic stress genes [81]. Phenylpropanoid metabolism has been identified as a source of tandemly repeated orthologous groups [81]. Based on their tight genetic linkage [83], *bona fide *CCoAOMT genes in conifers might be tandemly repeated in the genome. In this study we found that all three genes present in the spruce genome are functionally diverged. Thus, in multi-gene families of the phenylpropanoid/lignin pathway individual members can be recruited for plant development and pest resistance, respectively. Neo- and subfunctionalizations in such gene families can be important resources for genetic variation in the perennial. Recently, it was shown that members of the small *PAL *gene family in the herbaceous annual *Arabidopsis *can have both distinct and overlapping functions in processes related to growth, development, and responses to environmental stresses [90]. Our results add to the existing literature on the role of the phenylpropanoid pathway in the evolution of conifer defense mechanism against insect pests. Our findings highlight that specific genes within the phenylpropanoid pathway can be duplicated and diversified in long-lived conifers in a process that is fundamentally different from shorter lived angiosperm species.

## Methods

### Picea Glauca (Moench) Voss × Picea Engelmanii Parry ex Engelm. Pedigree

An outline of the experimental setup is given in Figure 2. Experimental spruce populations were chosen from a controlled-cross progeny trial established in 1995 at Kalamalka Research Station in Vernon, BC, Canada [43]. The respective males and females originated from individuals previously ranked for weevil-resistance in open-pollinated progeny tests [91]. Out of twenty crosses i.e., resistant-female-by-susceptible-male crosses, four crosses with markedly intermediate weevil resistance [43] and forming a partial diallel were studied in depth (cross 26 from ♀PG87*♂PG165, cross 27 from ♀PG87*♂PG117, cross 29 from ♀PG21*♂PG165 and cross 32 from ♀PG21*♂PG117, respectively and Figure 2), see also below.

In-silico SNPs were identified from the Treenomix EST database (K. Ritland pers. comm.). From these SNPs, a mapping population of 417 individuals involving a factorial cross design of 3 × 2 crosses was screened using a 384-plex GoldenGate Genotyping BeadArray Illumina platform ([92], Illumina Inc., San Diego, CA, USA) at the CMMT Genotyping and Gene Expression Core Facility, Centre for Molecular Medicine and Therapeutics, Vancouver, BC. Total genomic DNA had been isolated from flushing bud/needle tissue of those individuals according to the cetyltrimethylammonium bromide (CTAB) method established by [93]. Genotypes were scored using the BeadStudio software.

Of all putative SNP loci in the genotyping assay, 73.4-76.0% high quality SNPs were considered in the analysis; Individuals in the crosses that could not be confirmed as full-sibs were removed from subsequent phenotyping (cross 26: 7%, cross 27: 10%, cross 29: 4%, cross 32: 1% of the trees alive in 2006). Using a joint likelihood method [94] we determined recombination rates between each pair of loci, and relative genetic distances (centiMorgans) using JoinMap 3.0 software [95] (original genotype data are provided in the Additional File 3). We followed the approach by [96] for grouping markers and marker ordering. We included two additional crosses (C. Liewlaksaneeyanawin, pers. comm.,) in the analysis to maximize the number of mapped loci. The established framework genetic map is based on 252 SNPs and is presented in Additional File 2.

### Tree Height, Weevil Attack, and Oviposition Data

Measures were taken for the initial tree height in 1995 (year one), and heights in years three and five as well as leader length in year five preceding the artificial augmentation of the local weevil population in October of the same year (hgt1995, hgt1997, hgt1999, and ldr1999, respectively). Attack rates in 2000 and 2001 (atk2000, atk2001) were classified as successful 'top kills', 'failure' to kill the leader and 'no attack' [43], in addition, for the same years, egg counts along the leaders (egg2000, egg2001) were summarized into five discrete classes (successful egg laying was recognized as feeding punctures that contain egg covering fecal plugs), R. Alfaro, pers. Communication. For details see Additional File 4. In addition, the sum of weevil attacks and the sum of oviposition for 2000 and 2001 were used as traits (sum_atk, and sum_egg).

### Tissue Collection, RNA Preparation, and Microarray

Tree material within a replicate block was sampled in a randomized fashion among the plots (i.e., crosses, Figure 2). Terminal leaders from trees in a block were collected in the mornings of May 16, 17, and 18, 2006, respectively. At the location bark/phloem tissue was immediately harvested from cut leaders as described previously ([97,59]), flash frozen in liquid nitrogen, and stored at -80°C until processed. Total RNA from unattacked individuals was isolated following the protocol of [98] and quantified using NanoDrop^® ^ND-1000 Spectrophotometer; RNA integrity was evaluated using the Agilent 2100 Bioanalyzer. The 21,840 spruce ESTs on the array involved elements from 12 different cDNA libraries, built from different tissues (bark, phloem, xylem), which were under different developmental stages, as well as wound/methyljasmonate treated (ca. 6,500 elements) and untreated (ca. 15,400). Complete details of cDNA microarray fabrication and quality control are described elsewhere (S. Ralph and co-workers, Gene Expression Omnibus database GEO: GPL5423 and http://www.treenomix.ca/).

### Microarray Experimental Design, Gene Expression Profiling, and Pre-processing of Expression Data

After we tested six genotyped crosses using the previously collected phenotypic data (see above), we determined that genotype differences between most and least resistant progeny were highest in crosses 26, 27, 29, and 32. A distant pair design for microarray analysis that maximized direct comparisons between different alleles at each locus was originally introduced by [41] and was modified here for outbred individuals. We estimated the genetic distance for possible probe-pairs genome-wide by using all segregating SNP loci (122 on average). Such a procedure maximized the number of distant pairs in a given cross. A 25% improvement over random pairing was achieved. We also balanced the two dyes across the three replicate blocks (i.e., sampling on three different days), the different batches of microarray fabrication, and different experimenters (see below). Our design resulted in 94 hybridizations profiling 48 individuals in cross 26, 36 in cross 27, and 50 in cross 29, as well as 54 individuals in cross 32 (Additional File 5).

Hybridizations were performed using the Genisphere Array350 kit (Genisphere, Hatfield, USA) following manufacturer's instructions. Forty micrograms total RNA was reverse transcribed using Superscript II reverse transcriptase (Invitrogen) and oligo d(T18) primers with a 5' unique sequence overhang specific to either the Cy3 or Cy5 labeling reactions. The RNA strand of the resulting cDNA:RNA hybrid was hydrolyzed in 0.075 M NaOH/0.0075 M EDTA at 65°C for 15 min followed by neutralization in 0.175 M Tris-HCl (pH 8.0).

Following pooling of the appropriate Cy3 and Cy5 cDNAs, samples were precipitated with linear acrylamide and resuspended in 17.7 μL nuclease-free water. A 27.3 μL hybridization mixture containing 22.5 μL 2 × SDS buffer, 4 μL LNA d(T) blocker, 2 μg sheared salmon testes DNA (Invitrogen) and 0.3 μL of Cy5-labeled GFP cDNA (Cy5-dUTP and Ready-To-Go labeling beads, Amersham Pharmacia Biotech) was added to the cDNA probe. Immediately prior to use, arrays were pre-washed 2× in 0.1% SDS at room temperature for 5 min each, followed by two washes in MilliQ-H2O for 2 min each, 3 min at 95°C in MilliQ-H2O, and dried by centrifugation (3 min at 2000 rpm in an IEC Centra CL2 centrifuge with rotor IEC 2367-00 in 50 mL conical tube). The cDNA probe was heat denatured at 80°C for 10 min, then maintained at 65°C prior to adding to a microarray slide heated to 55°C, covered with a 22 × 60 × 1.5 mm glass coverslip (Fisher Scientific), and incubated for 16 h at 60°C. Arrays were washed in 2× SSC, 0.2% SDS at room temperature for 5 min to remove the coverslip, followed by 15 min at 65°C in the same solution, then three washes of 5 min in 2× SSC at room temperature, and three washes of 5 min in 0.2× SSC at room temperature, and dried by centrifugation. The Cy3 and Cy5 3DNA capture reagent (Genisphere) were then hybridized to the bound cDNA on the microarray in a 45 μL volume consisting of 22.5 μL 2× SDS buffer, 17.5 μL nuclease-free water, 2.5 μL Cy3 capture reagent, and 2.5 μL Cy5 capture reagent. The 3DNA capture reagent is bound to its complementary cDNA capture sequence on the Cy3 or Cy5 oligo d(T) primers. The second hybridization was performed for 3 h at 60°C and was then washed and dried as before.

Fluorescent images of hybridized arrays were acquired by using ScanArray Express (PerkinElmer, Foster City, USA). The Cy3 and Cy5 cyanine fluors were excited at 543 nm and 633 nm, respectively. All scans were performed at the same laser power (90%), but with the photomultiplier tube settings for the two channels adjusted such that the ratio of the mean signal intensities was ~1, and the percentage of saturated array elements was < 0.5% but > 0%, while minimizing background fluorescence. Fluorescent intensity data were extracted by using the ImaGene 6.0 software (Biodiscovery, El Segundo, USA). Signal intensity measurements were deposited in GEO under the accession number GSE22116.

After quantification of the signal intensities in each array were completed, the local background was subtracted for each subgrid. Data were normalized to compensate for non-linearity of intensity distributions using the variance stabilizing normalization method [99]. Finally, all 94 slides underwent simultaneous normalization to achieve a similar and array-independent overall expression level and variance for every channel. The linear model

(1) $${\text{h}}_{\text{i}}=\mu +\text{ dye }+\text{ block }+\text{ batch }+\text{ person }+\varepsilon_{\text{i}},$$

with *μ *as the overall mean, was then fit to the normalized intensities of each gene i (*h_i_*) in the Cy3 and Cy5 channels to account for technical effects within the experiment (gene-specific 'dye' effect, replicate 'block', microarray fabrication 'batch', experimenter 'person' are all fixed effects). The residuals were used in the subsequent eQTL analysis. The above statistics were carried out using the R statistical package http://www.r-project.org.

### QTL Analysis

QTLs were mapped in the diallel progeny by employing a likelihood function to assess the phenotype effect (gene expression, other traits) conditional on genotypic variation. We used R http://www.r-project.org to display QTL density maps. A QTL was significant at LOD≥3.84 (defined as the log likelihood ratio for the statistical support of the presence of a QTL within a 10 cM marker interval) and had to be detected for at least one parent in the diallel, however we did not show whether that specific QTL was also detected in any other parent (Figure 5). A goodness-of-fit test assuming a uniform distribution was performed to test whether the observed frequencies of eQTLs along the linkage map differed significantly from the expected value. Following the rejection of the null hypothesis (χ^2 ^= 3,551, df = 251, p-value < 2.2e-16), we declared "eQTL hotspots" if the number of eQTLs at a given locus was equal or above the maximum value (i.e., 38) for assessed eQTL clusters from a randomly generated data set using 4,221 eQTLs, 252 markers, and running 1,000 replicates. Positional candidate genes were identified by collocation of at least 40% of their eQTLs with phenotypic trait QTLs based on the criteria for identifying significant QTLs (see above) and running 10,000 randomizations (p ≤ 0.05).

### Co-Expression Network Analysis

We used the software package GeneNet 1.2.3 http://strimmerlab.org/software/genenet/ to assess gene-gene interactions. First, the 'family' effect was removed from the data using the ANOVA model

(2) $${\text{h}}_{\text{i}}=\mu +\text{ dye }+\text{ block }+\text{ family }+\text{batch}+\text{person }+\varepsilon_{\text{i}}.$$

The residuals were retained and utilized in the analysis. Gene co-expression based on partial correlations was assessed using a graphical Gaussian model (GGM) and a shrinkage method implemented in the GeneNet software package [100]. Here, neighbors have direct dependencies, since indirect gene connections are removed by this method. As a significance cut-off we used the 80% probability for presence of gene-pair interconnection. For visual representation of the network we used Pajek 1.24 software [101].

### Phylogenetic Analyses

Contigs for candidate genes (Figure 1) from white spruce (*Picea glauca*) were created *in silico *from EST sequences retrieved through reciprocal BLAST searches as described in [25-29]. An arbitrary cut-off of sequence identity exceeding 99% was used to identify possible allelic variation. Phylogenetic analyses on aligned amino acid sequences (dialign2; http://bioweb2.pasteur.fr/; manually curated; Bioedit v7.0.9 http://www.mbio.ncsu.edu/BioEdit/bioedit.html) were tested for consistency and the reconstructed maximum likelihood tree was bootstrapped [PhyML; http://www.atgc-montpellier.fr/phyml/binaries.php; four rate substitution categories, γ shape parameter optimized, JTT (Jones-Taylor-Thornton) substitution model, BioNJ starting tree and 100 bootstrap repetitions [102]] and displayed as phylogram using treeview32 1.6.6 (http://taxonomy.zoology.gla.ac.uk/rod/treeview.html[103]). Tree topologies were supported using the independent maximum likelihood algorithm TREE-PUZZLE 5.2 (http://www.tree-puzzle.de[104]). Array elements were mapped onto the phylogenetic trees by homology. Information about sequences from Arabidopsis and functionally characterized orthologues that were retrieved from public databases is given in Additional File 14. In decreasing order of priority, we used: (i) model plants with sequenced genomes (Arabidopsis, poplar and rice), (ii) functionally characterized members from non-model plants (gymnosperms, finally illustrative members from other angiosperms). Fully sequenced genomes allow assessing possible species- or lineage-specific expansions which are indicative of the mode of evolution (for example in the rice and spruce COMT superfamily). Functionally characterized individual members demonstrate diversification (neofunctionalization) in subfamilies.

## List of abbreviations

Picgl: *Picea glauca*; Picsi: *Picea sitchensis*; Pge: *Picea engelmanii*; 4CL: 4-coumarate-CoA ligase; ADT/PD: arogenate dehydratase/prephenate dehydratase; C4H: cinnamate-4-hydroxylase; CAD: cinnamyl-alcohol dehydrogenase; CCoAOMT: caffeoyl-CoA 3-O-methyltransferase; CCR: cinnamoyl-CoA reductase; CHI: chalcone (flavanone) isomerase; CHS: chalcone synthase; CM: chorismate mutase; CS: chorismate synthase; DAHP: 3-deoxy-D-arabino-heptulosonate 7-phosphate synthase; DFR: dihydroflavonol 4-reductase; DHQD-SD: 3-dehydroquinate dehydratase/shikimate 5-dehydrogenase; DHQS: 3-dehydroquinate synthase; DIR: disease resistance-responsive/dirigent; EPSPS: 5-enolpyruvylshikimate-3-phosphate/EPSP synthase; F3'H: flavonoid 3'-monooxygenase; F5H: ferulate 5-hydroxylase; FLS: flavonol synthase; LAC: laccase; OMT: O-methyltransferase; OPCL: oxo-pentenyl-cyclopentane ligase, PAL: phenylalanine ammonia-lyase; PCBER: phenylcoumaran benzylic ether reductase; PLR: pinoresinol-lariciresinol reductase; PRXR: peroxidase; SK: shikimate kinase; SAMS: S-adenosyl-methionine synthase; MYB: myeloblastosis domain; NAC: nascent polypeptide-associated complex; QTL: quantitative trait locus; eQTL: gene expression QTL; pQTL: phenotypic trait QTL; SNP: single nucleotide polymorphism; GGM: graphical Gaussian model; LOD: logarithm of the odds gives the likelihood ratio between two hypotheses.

## Authors' contributions

IP, RW, and KR designed experiments, conducted the data analysis and interpretation of data and results. IP carried out experiments. BH contributed to phylogenetic analysis. KR conceived of the overall study. IP and BH wrote the manuscript. All authors read and approved the final manuscript.

## Supplementary Material

Additional file 1 **List of 428 spruce candidate genes of the phenylpropanoid biosynthesis and related pathway from our microarray experiment, with AGI annotations, and trait associations**. Additional information is provided regarding the code of each individual transcript (microarray element) within the gene co-expression network (Figure 4) as well as about amino acid length and isoelectric point (pI) for the classification of the classIII peroxidases (prxr). Click here for file

Additional file 2 **Framework linkage map as displayed in **Figure 5, **252 SNPs, with annotations of contig sequences that were used for SNP detection**. Information about SNP loci annotation (Viridiplantae and TAIR7) and their positions along the linkage groups (LG) in centiMorgan map distance. Click here for file

Additional file 3 **Genotype data for individuals of the QTL mapping population with resolved sibship (see Material and Methods)**. SNPs that are part of the framework linkage map are displayed regarding their assignment to linkage groups (LG) and their positions on LGs in map distances (centiMorgan, cM). Click here for file

Additional file 4 **The phenotypic data for the four crosses 26, 27, 29, and 32 used in the eQTL study contain information about (a) tree height, (b) weevil attack, and (c) oviposition**. For explanations of column headers and codes, see end of table. Click here for file

Additional file 5 **Design and hybridizations as indicated in Methods and from which signal intensity measurements were deposited in GEO**. Information is provided for each hybridization (slide number as deposited in GEO) about the origin of the slide (batch identifiers A, B, C), the experimenter, i.e., the person identifier (1, 2, 3) for probe hybridization, the dye label of each RNA sample (Cy3 or Cy5) and the sample identifier (26A04, e.g., indicates an individual from cross26, replicate block A, plot tree 4). Click here for file

Additional file 6 **QTLs for (a) tree height, (b) weevil attack, and (c) oviposition. A QTL was significant at LOD≥3.84; allele effect and % phenotypic variation explained by QTL is given**. We summarized results for an expanded mapping population (five parents, a progeny of 369 individuals) as will be described elsewhere (Porth et al., in prep.) and for the following traits: tree heights (initial height in 1995, height in 1997, height in 1999), leader length in 1999, weevil attacks (assessed in 2000, in 2001 and the total attacks summed up for both years) and oviposition (egg plugs in 2000, in 2001, and total egg plugs summed up for both years). Click here for file

Additional file 7 **Significant QTLs for gene expression; allele effect and % phenotypic variation explained by the QTL is given**. Information about the detected eQTLs which were significant at LOD≥3.84 is provided including the information if a QTL was detected in more than one parent (these details are not shown in the density map of Figure 5), see Methods for further explanation. Click here for file

Additional file 8 **Phenylpropanoid P450 (F5H-F3H), phylogenetic tree, array elements (in red)**. Click here for file

Additional file 9 **Eight phenotype associated expression hotspots with listings of gene family members that contributed eQTLs (gene annotations where applicable)**. See also the information given in Additional File 1 and Figure 5. Click here for file

Additional file 10 **Summary on gene families with individual members having eQTLs at a minimum of two phenotype associated expression hotspots**. This table summarizes characteristics of the investigated gene families associated with the phenylpropanoid pathway and shows the total number of gene-family members, the detected average number of eQTLs per family, and the number of the identified gene-family members with eQTLs at a minimum of two out of the eight identified regulatory hotspots as shown in Figure 6. Click here for file

Additional file 11 **The O-methyltransferase superfamily family including outgroup PicglOMT1, array element (in red)**. Click here for file

Additional file 12 **Catechol-OMTs including ancient white spruce Catechol-O-Methyltransferase (PicglOMT2), array element (in red)**. Click here for file

Additional file 13 **Class III Peroxidases, phylogenetic tree, array elements (in red) and non-treenomix, phyml 10× tree, rooted with Spruce67/86**. Click here for file

Additional file 14 **Accessions and identifiers of non-spruce members of the phenylpropanoid pathway families**. Accessions and identifiers are provided for the following gene families: OMT/OMTL, CCoAOMT, PCBER, PKS, 4CL/ACL, and ADT. Click here for file
