# Supplementary figures and images for: Defense mechanisms against herbivory in Picea: sequence evolution and expression regulation of gene family members in the phenylpropanoid pathway
Source: BMC Genomics. 2011 Dec 16;12:608. doi: 10.1186/1471-2164-12-608 (PMC3288119; doi:10.1186/1471-2164-12-608)

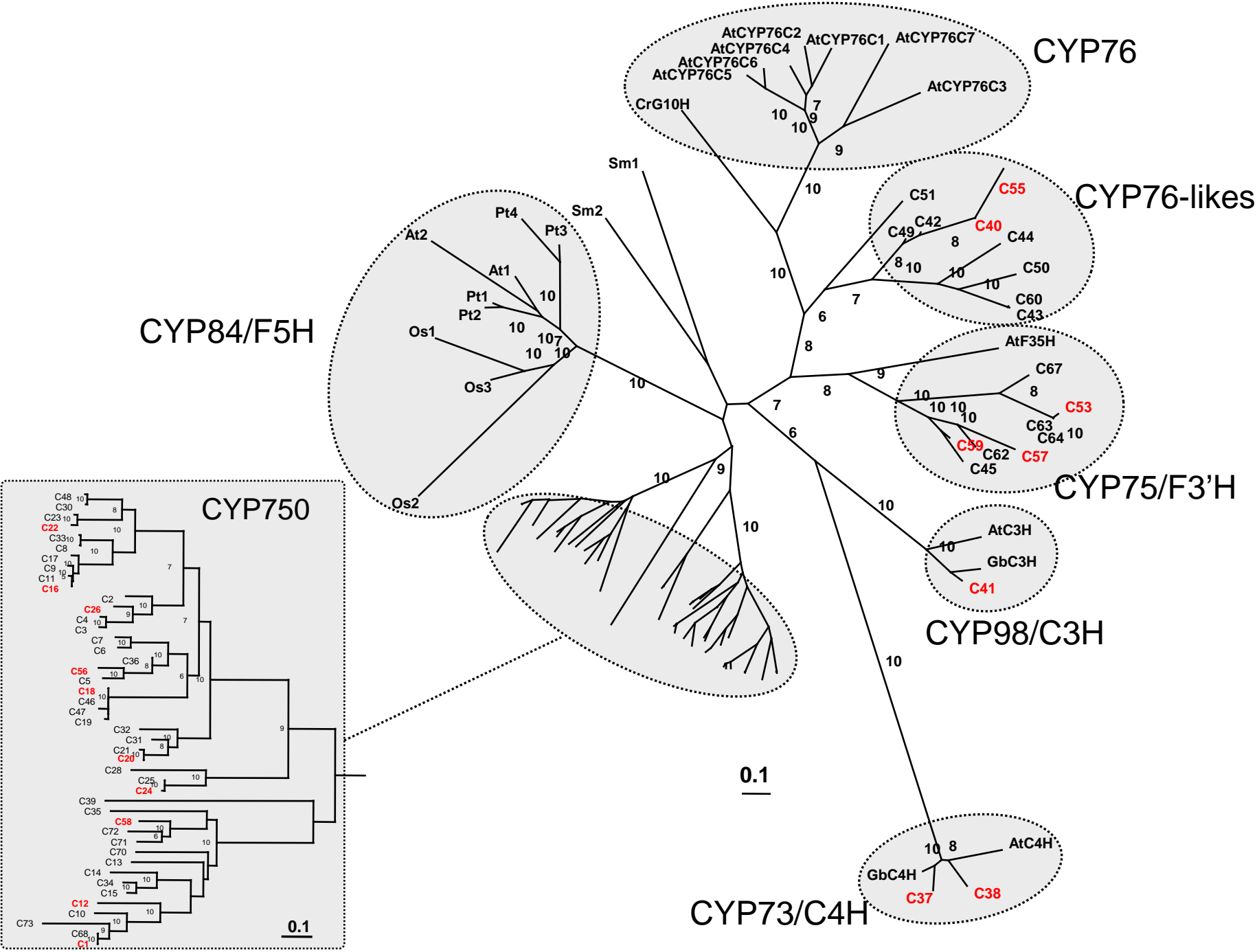

Supplement: Additional file 8 — Phenylpropanoid P450 (F5H-F3H), phylogenetic tree, array elements (in red). [file 1471-2164-12-608-S8.PDF]

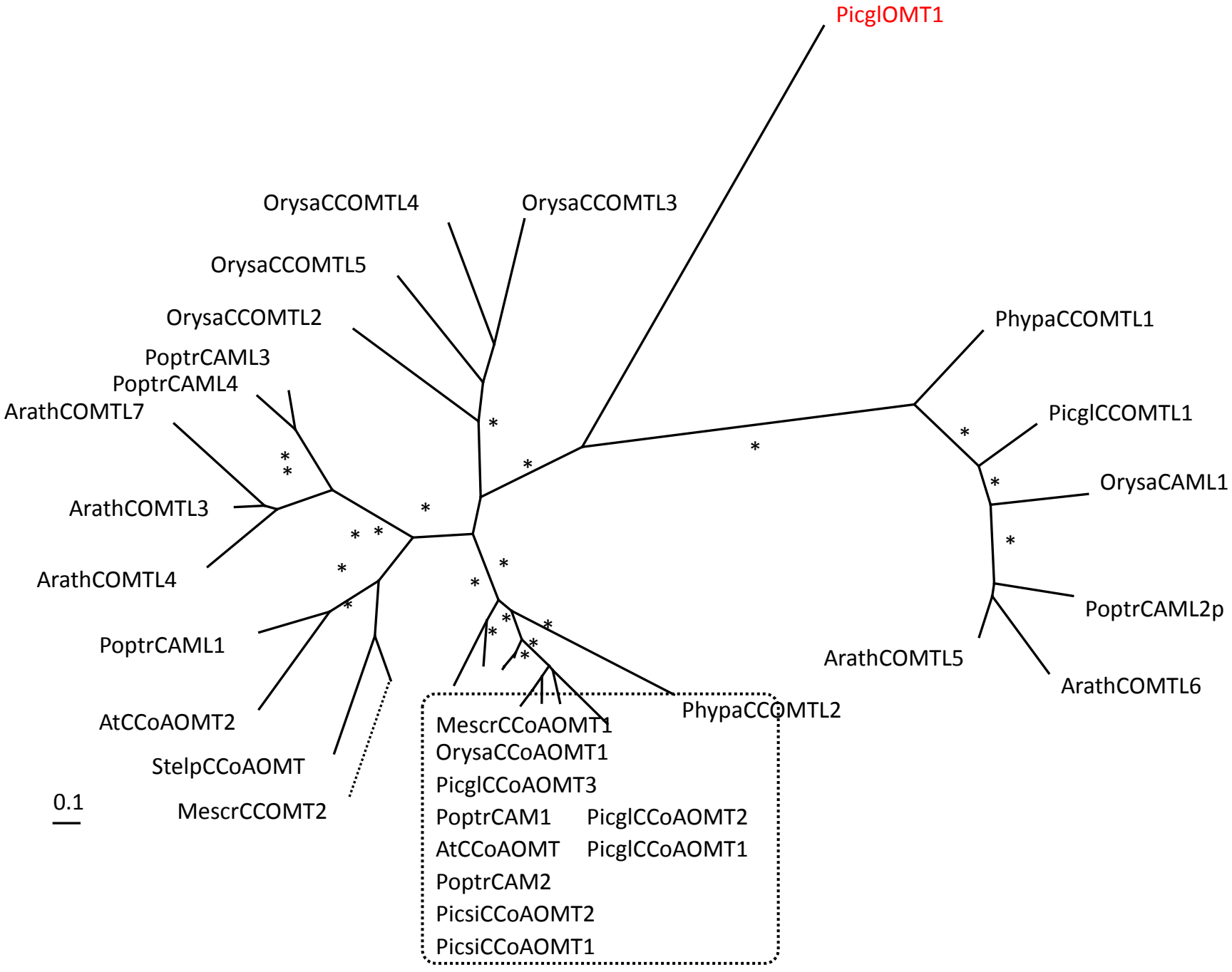

Supplement: Additional file 11 — The O-methyltransferase superfamily family including outgroup PicglOMT1, array element (in red). [file 1471-2164-12-608-S11.PDF]

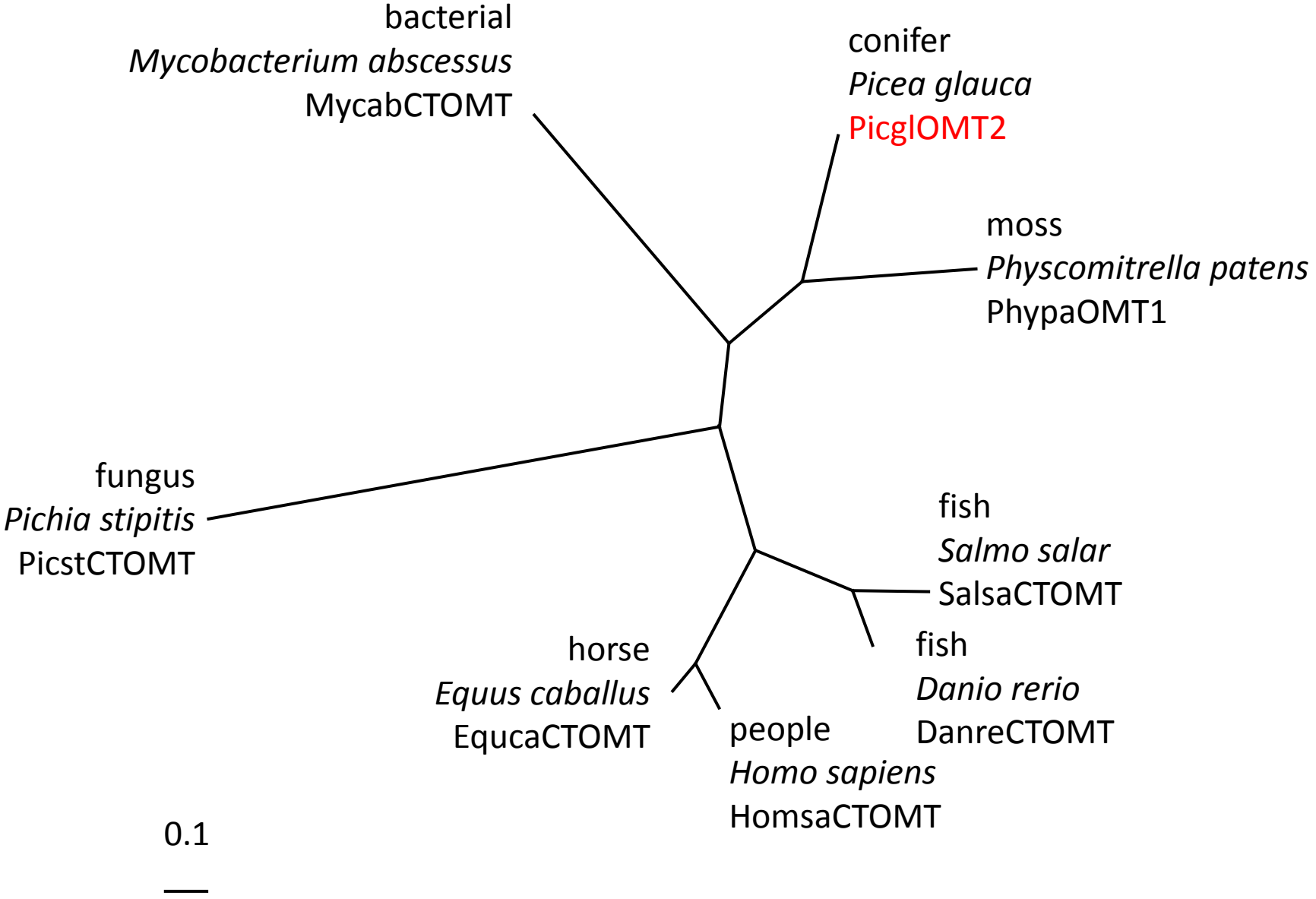

Supplement: Additional file 12 — Catechol-OMTs including ancient white spruce Catechol-O-Methyltransferase (PicglOMT2), array element (in red). [file 1471-2164-12-608-S12.PDF]

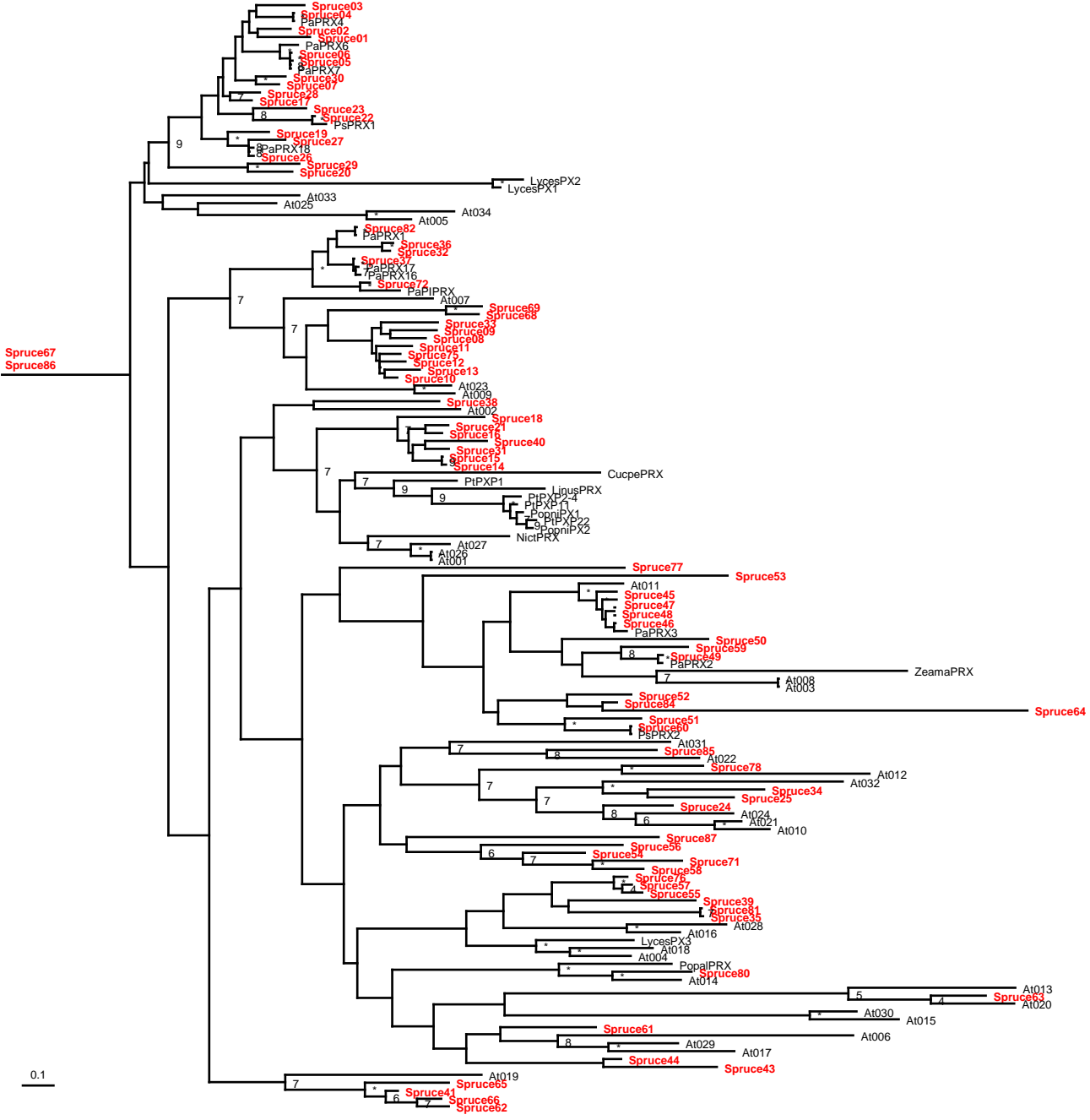

Supplement: Additional file 13 — Class III Peroxidases, phylogenetic tree, array elements (in red) and non-treenomix, phyml 10× tree, rooted with Spruce67/86. [file 1471-2164-12-608-S13.PDF]
